# A role for 3′-O-β-D-ribofuranosyladenosine in altering plant immunity

**DOI:** 10.1016/j.phytochem.2018.10.016

**Published:** 2019-01

**Authors:** Mikhail S. Drenichev, Mark Bennett, Roman A. Novikov, John Mansfield, Nick Smirnoff, Murray Grant, Sergey N. Mikhailov

**Affiliations:** a Engelhardt Institute of Molecular Biology, Russian Academy of Sciences, Vavilov Str. 32, Moscow, 119991, Russian Federation; b Imperial College London, Exhibition Road, London, SW7 2AZ, United Kingdom; c School of Biosciences, University of Exeter, Stocker Road, Exeter, EX4 4QD, United Kingdom; d School of Life Sciences, Gibbet Hill, University of Warwick, Coventry, CV4 7AL, United Kingdom

**Keywords:** Disaccharide nucleosides, *Arabidopsis thaliana*, *Pseudomonas syringae*, Phytopathogens

## Abstract

Our understanding of how, and the extent to which, phytopathogens reconfigure host metabolic pathways to enhance virulence is remarkably limited. Here we investigate the dynamics of the natural disaccharide nucleoside, 3′-O-β-D-ribofuranosyladenosine, in leaves of *Arabidopsis thaliana* infected with virulent *Pseudomonas syringae* pv. tomato strain DC3000. 3′-O-β-D-ribofuranosyladenosine is a plant derived molecule that rapidly accumulates following delivery of *P. syringae* type III effectors to represent a major component of the infected leaf metabolome. We report the first synthesis of 3′-O-β-D-ribofuranosyladenosine using a method involving the condensation of a small excess of 1-O-acetyl-2,3,5-three-O-benzoyl-β-ribofuranose activated with tin tetrachloride with 2′,5′-di-O-tert-butyldimethylsilyladenosine in 1,2-dichloroethane with further removal of silyl and benzoyl protecting groups. Interestingly, application of synthetic 3′-O-β-D-ribofuranosyladenosine did not affect either bacterial multiplication or infection dynamics suggesting a major reconfiguration of metabolism during pathogenesis and a heavy metabolic burden on the infected plant.

## Highlights

• We describe the first synthesis of 3′-O-β-D-ribofuranosyladenosine.
• 3′-O-β-D-ribofuranosyladenosine is a natural disaccharide nucleoside induced by phytopathogens.
• 3′-O-β-D-ribofuranosyladenosine is plant derived.
• 3′-O-β-D-ribofuranosyladenosine accumulates less in *A. thaliana* hyper-resistant mutants.
• The amount of 3′-O-β-D-ribofuranosyladenosine can accumulate to ∼1.5 μg per infected leaf.

## Graphical abstract

**Figure:**
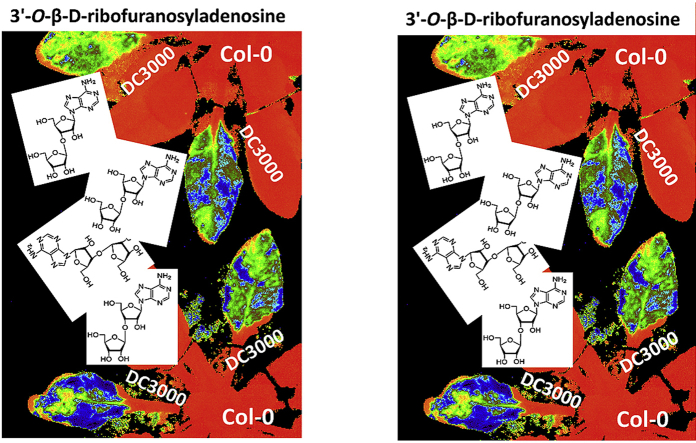

## 1 Introduction

Our understanding of the genetic basis of plant innate immunity has improved greatly over the past two decades. Biochemically, we understand the core receptors involved in the two key immunity responses, the initial MAMP (microbe associated molecular patterns) triggered immunity (MTI) and subsequent effector triggered immunity (ETI) where activity of pathogen effectors delivered into the plant cell are recognized by classical plant disease resistance proteins (Jones and Dangl, 2006). We are unravelling the basis of suppression of plant immunity, driven by pathogen effectors (primarily proteins, but also small molecules), which suppress MTI and ETI to cause disease (effector triggered suppression, ETS; (Boutrot and Zipfel, 2017; Jones and Dangl, 2006)). While our understanding of the underlying transcriptional reprogramming association with this transition from defense to disease is becoming clearer (Lewis et al., 2015), our knowledge of the small molecules deployed to effect this transition is notably limited.

About fifteen years ago an exciting discovery reported that phytopathogenic bacteria such as the Pseudomonadaceae *Pseudomonas syringae*, induced the formation of a novel disaccharide nucleoside 3′-O-β-D-ribofuranosyladenosine in infected leaves (Bednarek et al., 2004). Subsequently, untargeted approaches validated 3′-O-β-D-ribofuranosyladenosine as a key discriminant molecule of a compatible interaction between the model Brassicaceae *Arabidopsis thaliana* and the virulent *Pseudomonas syringae* pv. tomato strain DC3000 (DC3000) (Ward et al., 2010). 3′-O-β-D-ribofuranosyladenosine (hereafter referred to as 3′-O-β-D-RFA) is a particularly interesting molecule, not only for its unique structure, but also because to date most specialised metabolites associated with *Arabidopsis thaliana* responses to pathogens are predominately indolic derivatives. The rapid induction of 3′-O-β-D-RFA to high levels upon infection in both tomato and *A. thaliana* precedes reported increases in indole derivatives (Bednarek et al., 2004; Ward et al., 2010) and suggests an important role in suppression of plant immunity.

Disaccharide nucleosides belong to an important group of natural compounds, forming components of biopolymers, such as poly(ADP-ribose) and tRNA, which underpin fundamental roles in living organisms (Drenichev & Mikhailov, 2015, 2016; Efimtseva et al., 2009; Efimtseva and Mikhailov, 2002). These compounds contain an extra carbohydrate residue linked to one of the nucleoside hydroxyl groups *via* an *O*-glycosidic bond. The presence of a disaccharide residue and a heterocyclic base makes their properties similar to those of carbohydrates and nucleosides (Drenichev & Mikhailov, 2015, 2016; Efimtseva et al., 2009; Efimtseva and Mikhailov, 2002). Over one hundred such compounds and their derivatives have been isolated from various sources and are implicated in a broad spectrum of biological activities, including antibacterial, fungicidal, herbicidal, anti-tumour and antiviral (Drenichev & Mikhailov, 2015, 2016; Efimtseva et al., 2009; Efimtseva and Mikhailov, 2002).

There is increasing evidence that ATP, NAD and PARP mediated ADP-ribosylation play a role in plant immune responses (see (Miwa et al., 2017; Petriacq et al., 2013) for reviews). Originally described as a mechanism of DNA break repair, poly(ADP-ribos)ylation has been linked to transcriptional control of gene expression, regulation of metabolism and dynamic reorganization of chromatin structure. In plants poly(ADP-ribos)ylation has received surprisingly little attention. It has been implicated in cycle control, development and response to abiotic and biotic stress. Mutations in genes encoding poly-ADPribosylases (*parps*; (Adams-Phillips et al., 2010; Adams-Phillips et al., 2008; Song et al., 2015)) or NUDIX ADP-ribose/NADH pyrophosphohydrolases (Ge et al., 2007; Ishikawa et al., 2010; Jambunathan et al., 2010) affect basal immunity, whereas production of NAD+ derivatives through metabolism of quinolate in plants expressing *E. coli nadC* enhanced resistance to already strong ETI interactions (Petriacq et al., 2012). The pyridine nucleotides NAD+ and NADP + play vital roles in metabolic reactions, either as signal molecules themselves or *via* their derivatives. Indeed, they are being increasingly linked to plant immune processes (see (Miwa et al., 2017; Petriacq et al., 2012) for reviews). Consistent with this, multiple mutations in aspartate oxidase, the chloroplast localized primary enzyme of *de novo* NAD+ synthesis (Katoh et al., 2006), were identified in a genetic screen for compromised basal immunity to DC3000 (Macho et al., 2012). Enhanced resistance was also shown to be associated with increased pools of intracellular NAD+ in quinolate treated *nadC* plants, correlating ETI resistance with enhanced reactive oxygen species (ROS) which, unlike classical respiratory burst homologue (RBHO) derived apoplastic ROS, was of mitochondrial origin (Petriacq et al., 2016).

Taking into account the well-established role of ROS generation in plant innate immunity (both PTI and ETI), it is entirely feasible to speculate that effective plant pathogens may deploy strategies to reduce the pool of pyridine nucleotides as a core part of their ETS mechanism. Given both the novel and highly interesting compound class, and its rapid accumulation early in establishment of disease, we further investigated the role of 3′-O-β-D-RFA in plant-pathogen interactions.

## 2 Results

### 2.1 Accumulation of 3′-O-β-D-ribofuranosyladenosine during infection of plant leaves with *Pseudomonas syringae*

**Fig. 1:**
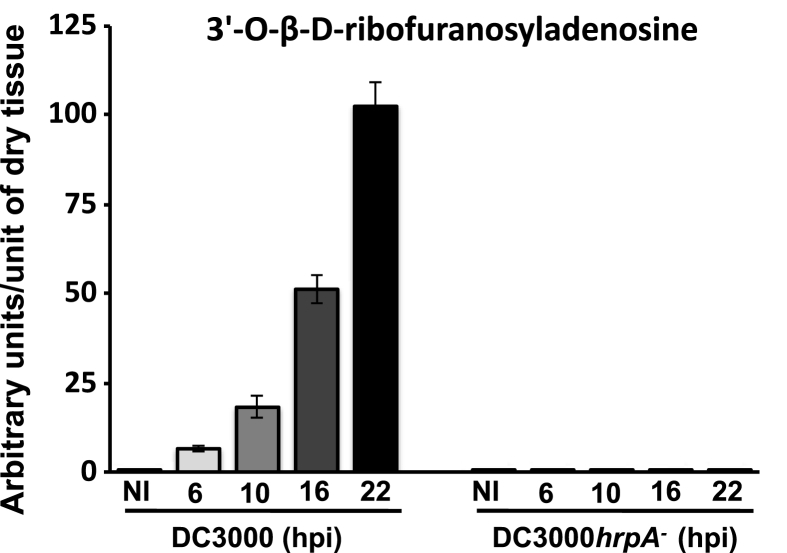
**Dynamics of foliar 3′-O-β-D-ribofuranosyladenosine accumulation following challenge with virulent and non-pathogenic *P. syringae***. Leaves of Col-0 wild type *A. thaliana* plants were challenged with either the virulent *P. syringae* DC3000 or the non-pathogenic DC3000*hrpA* mutant (OD_600_ = 0.15) and samples taken at the time shown. Metabolites were extracted in 10% methanol, 1% acetic acid and relative levels of 3′-O-β-D-ribofuranosyladenosine accumulation over time between the two treatments were determined. NI – non-induced. The figure is representative of three independent experiments, with error bars representing the standard deviation of the mean.

Using a single reaction monitoring method previously established using purified 3′-O-β-D-RFA standard (Ward et al., 2010) we monitored 3′-O-β-D-RFA accumulation in leaves following DC3000 infection. 3′-O-β-D-RFA accumulated remarkably rapidly, within 4 h post infection (hpi) with DC3000 (OD_600_ 0.15; Fig. S1) and continued to accumulate exponentially, mirroring the observed bacterial growth measured under identical conditions (Lewis et al., 2015). The rapid appearance of 3′-O-β-D-RFA was striking as bacterial effectors are not delivered into the plant cells until ∼3 hpi after challenge and DC3000 multiplication does not occur under those conditions until ∼8 hpi (Lewis et al., 2015). Bacterial growth is conditional on ETS accomplished by the collective actions of the bacterial effectors, a pre-requisite being suppression of both photosynthesis and chloroplast reactive species generation (de Torres Zabala et al., 2015). To determine the origin of 3′-O-β-D-RFA we compared its accumulation in uninfected leaves with leaves challenged with DC3000*hrpA* (this mutant strain cannot deliver effectors to suppress immunity and elicits a basal immune response), or virulent DC3000 (causes foliar disease). Uninfected leaves contain very low levels of 3′-O-β-D-RFA suggesting this was of plant origin (Fig. 1). No significant accumulation of 3′-O-β-D-RFA was detected following DC3000*hrpA* challenge, consistent with our hypothesis that the activity of the 28 DC3000 effectors delivered into the plant cell (Cunnac et al., 2011) were responsible for its accumulation. To rule out the possibility 3′-O-β-D-RFA is of bacterial origin we used a transgenic *A. thaliana* line conditionally expressing the *P. syringae* effector *HopAM1* from a dexamethasone inducible promoter in Wassilewskija background (Goel et al., 2008). Conditional expression of *HopAM1* increases *P. syringae* virulence by enhancing pathogen induced ABA that play a key role in suppressing plant defense responses. Leaves of a transgenic *A. thaliana* Ws-0 lines conditionally expressing *HopAM1* from a dexamethasone inducible promoter showed accumulation of 3′-O-β-D-ribofuranosyladenosine within 12 h of dexamethasone (5 μM) application. By contrast 3′-O-β-D-RFA did not accumulate above basal levels in wild type *A. thaliana* Ws-0 (Fig. S2).

**Fig. 2:**
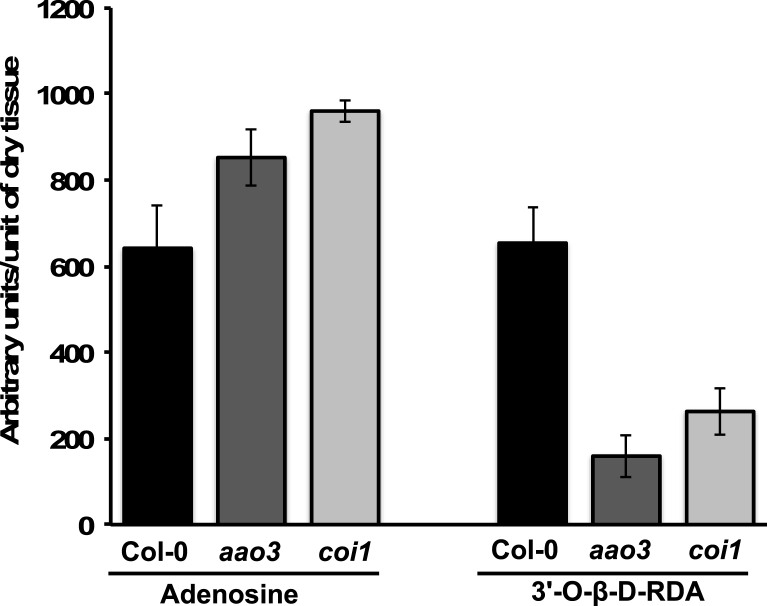
**3′-O-β-D-ribofuranosyladenosine appears to be derived from adenosine and accumulation is related to host susceptibility to *P. syringae* DC3000**. Both the ABA biosynthetic mutant *aao3*, that prevents pathogen accumulation of the immunosuppressor ABA and the jasmonate receptor mutant *coi1* are more resistant to DC3000. Both mutants exhibit higher levels of foliar adenosine and much reduced levels of 3′-O-β-D-ribofuranosyladenosine compared to susceptible wild type Col-0 following challenge with DC3000 (OD_600_ = 0.15) sampled 18 hpi. Error bars represent the standard deviation of the mean. This experiment was repeated twice.

To determine whether accumulation of 3′-O-β-D-RFA is genetically linked to DC3000 virulence we examined a broad spectrum of *Arabidopsis* mutants showing enhanced resistance to DC3000. DC3000 hijacks the phytohormone ABA signalling pathway to promote virulence (de Torres-Zabala et al., 2007) and the ABA biosynthetic mutant *Arabidopsis aldehyde oxidase 3* (*aao3*), deficient in the penultimate step of ABA biosynthesis, is more resistant to DC3000 infection (de Torres Zabala et al., 2009). Another phytohormone, jasmonic acid also suppresses plant defenses and *A. thaliana* mutants in the jasmonate receptor Coronatine Insensitive 1 (COI1) are more resistant to DC3000 (Brooks et al., 2005). In leaves of the *aao3* or *coi1 A. thaliana* mutants challenged with DC3000, 3′-O-β-D-RFA accumulated to significantly lower levels than wild type Col-0 leaves (Fig. 2) correlating 3′-O-β-D-RFA levels with severity of disease development. Moreover, consistent with the prediction that adenosine is a likely precursor for 3′- O-β-D-RFA synthesis, leaf adenosine levels were lower in DC3000 challenged leaves which accumulate more 3′-O-β-D-RFA than either of the two mutants (Fig. 2). The accumulation of high levels of 3′-O-β-D-RFA in infected leaves suggests a major reconfiguration of metabolism during pathogenesis and a heavy metabolic burden on the infected plant. Moreover, since these mutants affected quite distinct signalling pathways, these data suggest that 3′-O-β-D-RFA is a metabolite directly associated with disease progression, rather than being associated with a specific arm of DC3000's multifaceted virulence strategy.

### 2.2 Synthesis of 3′-O-β-D-ribofuranosyladenosine

**Fig. 3:**
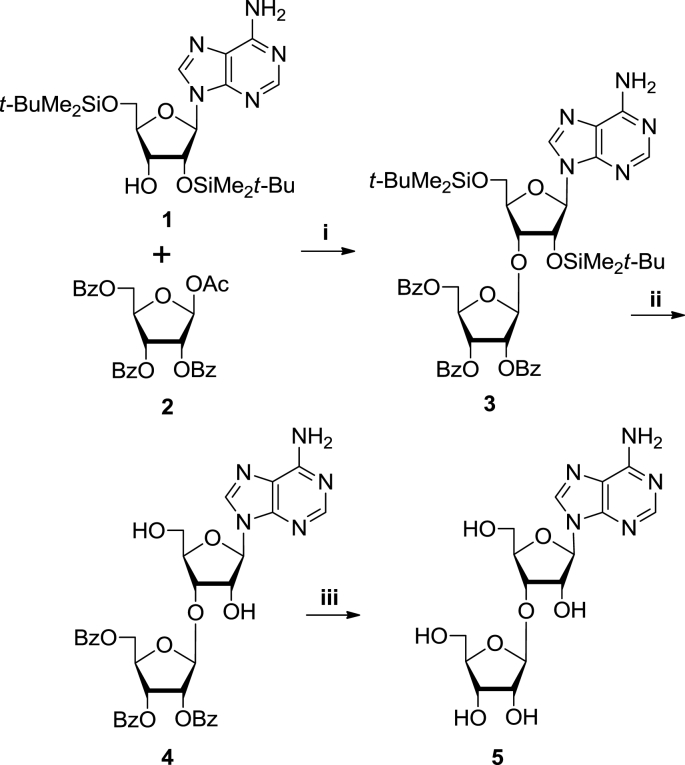
**Synthesis of 3′-O-β-D-ribofuranosyladenosine**. A three step procedure was developed in which; (**i)** The nucleoside 2′,5′-di-*O*-*tert*-butyldimethylsilyladenosine (**1**) was glycosylated with 1-*O*-acetyl-2,3,5-three-*O*-benzoyl-β-D-ribofuranose (**2**) in the presence of tin tetrachloride SnCl_4_/1,2-dichloroethane at 0 °C for 16 h generating blocked 3′-O-β-D-ribofuranosyl adenosine (∼64% efficiency); (**ii)** This was desilylated and partially debenzoylated in the presence of tetrabutylammonium fluoride trihydrate for 45 min, yielding 9-[3-*O*-(2,3,5-three-*O-*benzoyl-β-D-ribofuranosyl)-β-D-ribofuranosyl]adenine (**4**) at ∼55%; and finally (**iii) 4** was debenzoylated in methanolic ammonia to yield 3′-O-β-D-ribofuranosyladenosine (**5**) at ∼65% efficiency and this was subsequently purified by crystallization from water.

To determine the biological function of 3′-O-β-D-RFA, we designed a three step chemical synthesis strategy for 3′-*O*-β-D-ribofuranosyladenosine (Fig. 3). First we developed a general route for the preparation of 2ʹ-O-β-D-ribofuranosylnucleosides by condensation of *N*-acyl-3ʹ,5ʹ-O-(tetra-isopropyldisiloxane-1,3-diyl)-ribonucleosides with a slight excess of 1-*O*-acetyl-2,3,5-three-*O*-benzoyl-β-ribofuranose in the presence of tin tetrachloride in 1,2-dichloroethane at 0 °C (Mikhailov et al., 1995, 1997, 2005; Rodionov et al., 2000) *O*-Glycosylation proceeded stereospecifically with formation of a β-glycosidic bond. This method was used for the preparation of pyrimidine 3ʹ-O-β-D-ribofuranosyl-2ʹ-deoxyribonucleosides (Mikhailov et al., 1996). 5ʹ-O-β-D-ribofuranosyl-2ʹ-deoxyribonucleosides and 5ʹ-O-β-D-ribofuranosylnucleosides (Mikhailov et al., 1998).

In our first attempt for the synthesis 3′-O-β-D-RFA we used *N*^6^-benzoyl-2′,5′-di-*O*-*tert*-butyldimethylsilyladenosine as the glycosyl-acceptor. Unfortunately, a mixture of products was formed, probably due to the instability of the glycosidic bonds of purine nucleosides (Gulyaeva et al., 2004). Moreover, the product and the initial glycosyl-acceptor had comparable chromatographic mobility and thus were difficult to separate by chromatographic purification. Therefore, the protected nucleoside 2′,5′-di-*O*-*tert*-butyldimethylsilyladenosine (**1**) with its free amino group was chosen as a more suitable glycosyl-acceptor. Glycosylation of (**1**) with 1-*O*-acetyl-2,3,5-three-*O*-benzoyl-β-D-ribofuranose (**2**) in the presence of tin tetrachloride was stereospecific resulting in blocked 3′-O-β-D-ribofuranosyladenosine (**3**). This reaction (Fig. 3 step i) was carried out under mild conditions (0 °C; 1,2-dichloroethane; 16 h) as previously described (Mikhailov et al., 1995, 1997, 2005; Rodionov et al., 2000). The resulting product, **3,** was easily purified by column chromatography and was isolated with 64% yield. The choice of synthetic strategy, involving 2′,5′-di-*O*-*tert*-butyldimethylsilyladenosine (**1**) (Ogilvie et al., 1978) as a glycosyl-acceptor, is also associated with higher stability of the silyl protecting group to the migration in the 2′,3′-*cis*-diol nucleosidic system under glycosylation conditions compared to acyl groups.

We performed desilylation of nucleoside **3** in the presence of tetrabutylammonium fluoride trihydrate (Fig. 3, step ii). Partial debenzoylation also took place as a by-process due to the basic character of the fluoride ion. Therefore, the resulting 9-[3-*O*-(2,3,5-three-*O-*benzoyl-β-D-ribofuranosyl)-β-D-ribofuranosyl]adenine (**4**) was isolated with 55% yield. Debenzoylation of nucleoside **4** was performed in methanolic ammonia. The final product, 3′-O-β-D-ribofuranosyladenosine (**5**) was purified by crystallization from water (Fig. 3 step iii) with 65% yield resulting in an overall yield of 23%.

While the majority of the known disaccharide nucleosides demonstrate high solubility in water because of the presence of two hydrophilic carbohydrate moieties in their structure, interestingly compound **5**, bearing five hydroxyl groups and one amino-group, had low solubility in both water and organic solvents.

The ^1^H-NMR spectrum of the synthesized compound **5** was identical in all respects to the spectrum of 3′-O-β-D-RFA, isolated from natural sources (Table 1; See also Supplementary Data, Figs. S3–S19). The presence of the (1'→3′)-glycosidic bond was confirmed by heteronuclear multiple bond correlation (HMBC) NMR. In HMBC, the spectra of compounds **4** and **5**, the cross-peaks between H-1' (Rib) and С-3' (Ado) and between H-3' (Ado) and C-1' (Rib) are present (Figs. S10 and S17). The coupling constant for *trans*-orientated protons H-1' (Rib) and H-2' (Rib) in extra ribose residue is *J*_*1′,2'*_ < 0.5 Hz, that is characteristic for β-configuration of glycosidic bond in disaccharide nucleosides (Mikhailov et al., 1996, 1997). UV-spectrum of 3′-O-β-D-RFA in H_2_O is characterized by absorption maximum at 259 nm (*ε* ∼14900) at рН 7–13 with slightly hypsochromic shift under acidic conditions (Fig. S19), which is typical for adenosine derivatives (Albert, 1973; Dowson et al., 1986).

**Table 1:** ^1^H-NMR data for compound **5** in CD_3_OD.

Nucleoside:	Chemically synthesized^a^		Isolated from natural sources^b^	
Fragment	Ado^c^	β-Rib^c^	Ado^c^	β-Rib^c^
H-1' (*J*_*1′, 2′*_)	6.00 d (4.3)	5.07 s (<0.5)	6.00 d (4.3)	5.05 s (<0.5)
H-2' (*J*_*2′, 3′*_)	4.71 dd (5.2)	4.06 d (4.7)	4.70 dd (5.1)	4.04 d (4.5)
H-3' (*J*_*3′, 4′*_)	4.43 t (5.1)	4.37 dd (7.2)	4.43 t (5.1)	4.37 dd (7.2)
H-4' (*J*_*4′, 5′a*_)	4.23 ddd (2.4)	4.00 ddd (2.5)	4.21 ddd (2.3)	3.98 ddd (2.3)
H-5′a(*J*_*5′a, 5′b*_)	3.81 dd (−12.3)	3.92 dd (−12.7)	3.82 dd (−12.5)	3.92 dd (−12.5)
H-5′b (*J*_*4′, 5′b*_)	3.76 dd (2.8)	3.68 dd (3.1)	3.77 dd (2.8)	3.67 dd (3.1)
H-8	8.35 s		8.40 s	
H-2	8.20 s		8.21 s	

a NMR spectrum was obtained at 400 MHz.

b NMR spectrum was obtained at 500 MHz (Bednarek et al., 2004).

c s: singlet, d: doublet, dd: doublet of doublet, ddd: doublet of doublet of doublet, t: triplet.

### 2.3 Assessing the biological activity of 3′-O-β-D-ribofuranosyladenosine

**Fig. 4:**
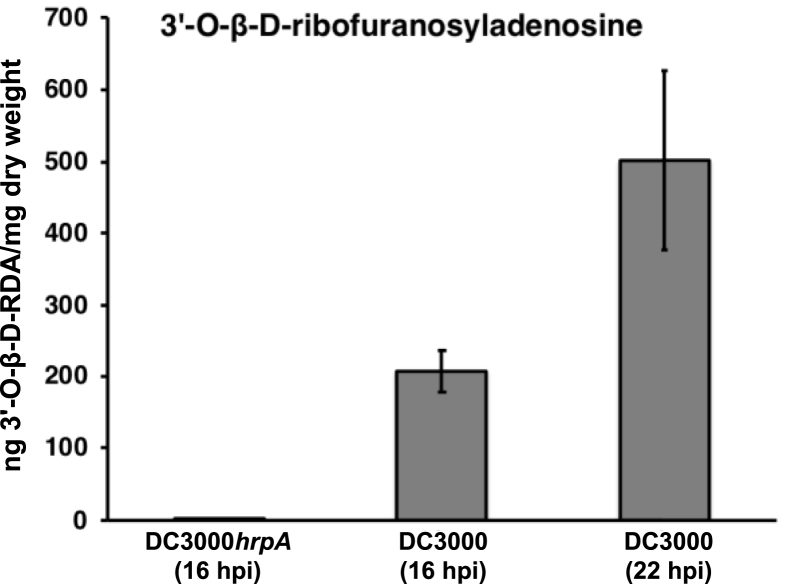
**Quantitation of 3′-O-β-D-ribofuranosyladenosine during *P. syringae* disease development**. Synthetic 3′-O-β-D-ribofuranosyladenosine standard was used to quantify pathogen induced 3′-O-β-D-RFA accumulation in leaves following challenge with DC3000 (OD_600_ 0.15). Error bars representing standard deviation of the mean. All experiments were repeated at least three times.

Following synthesis, we first determined the extent of accumulation of 3′-O-β-D-RFA following DC3000 infection. Fig. 4 shows that 3′-O-β-D-RFA represents ∼1/2000^th^ of the dry weight of an infected leaf 22 h after infection with DC3000 (OD_600_ 0.15). We next tested the impact of 3′-O-β-D-RFA on the infection process.

Based on an average leaf DW of 2.4 mg (Browse and Somerville, 1994), 3′-O-β-D-RFA levels were estimated to be ∼1.5 μg per leaf 22 h post infection. Therefore we either pre-infiltrated (16 h before infection) or co-infiltrated DC3000 with two synthetic 3′-O-β-D-RFA concentrations (50 μg/ml or 100 μg/ml) corresponding to delivery of 2 μg or 4 μg of 3′-O-β-D-RFA/leaf based upon a conservative average of 40μl/leaf of inoculum. Neither treatments resulted in any significant differences in bacterial growth (data not shown). Similarly, supplementation of 3′-O-β-D-RFA to Kings B plates (25 μg/ml) had neither a positive nor negative affect on DC3000 multiplication (data not shown). This lack of demonstrable biological activity associated with 3′-O-β-D-RFA, i.e. neither suppression or enhancement of immunity following co-infiltration, or alternatively, restriction or enhancement of DC3000 growth *in vitro*, was surprising given the remarkable rapid infection dynamics of this novel molecule following DC3000 inoculation. While we can rule out an antimicrobial effect of 3′-O-β-D-RFA on *P*. s*yringae*, we cannot determine from the *in vivo* studies that apoplastically3′-O-β-D-RFA delivered into the apoplast was taken up into the plant cells. An alternative possibility to consider is that 3′-O-β-D-RFA is actually an artefact of the extraction procedure and a highly related compound is actually the biologically active species formed by DC3000 infection. Future work will explore these possibilities.

## 3 Discussion

In summary, we comprehensively demonstrate the dynamics of plant derived 3′-O-β-D-RFA accumulation during susceptible interactions and present a detailed synthesis strategy, supported by NMR validation. It was unambiguously demonstrated that our synthetic 3′-O-β-D-RFA has the same chromatographic mobility with the cognate metabolite rapidly induced in DC3000 infected leaves. However, in the absence of any detectable biological activity on bacterial disease progression or host defense processes we hypothesise that 3′-O-β-D-RFA may represent a degradation product of a highly labile disaccharide nucleoside that is modified under our extraction conditions. Alternatively 3′-O-β-D-RFA's sole role may be to deplete the plant pool of pyridine nucleotides and suppress one arm of the plant immune response. Regardless, accumulation of a disaccharide nucleoside to such levels suggests major metabolic re-programming and is likely to have a significant impact on the plants metabolic status and warrants further investigation.

Mono ADP-ribosyltransferases cleave NAD^+^ and covalently attach the ADP-ribosyl moiety to target proteins, or DNA/RNA (Hassa et al., 2006). DC3000 has at least 4 predicted mono ADP-ribosyltransferases (Hop-U1, Hop-F2, HopO1-1, HopO1-2) that are annotated as effectors (Block and Alfano, 2011; Lindeberg et al., 2012) and could modify host proteins, DNA/RNA or, as we hypothesise here, small molecules leading to the generation of 3′-O-β-D-RFA. This raises the question as to what the 3′-O-β-D-RFA substrate(s) may be. The pyridine nucleotides NAD+ and NADP + play vital roles in metabolic reactions, as signal molecules themselves or *via* their derivatives and in plants are being increasingly linked to plant immune processes (Miwa et al., 2017). Given the well-established role of reactive oxygen generation in plant innate immunity (both PTI and ETI), one possibility is that plant pathogens may deploy strategies to reduce the pool of pyridine nucleotides to suppress plant defense responses. We recently showed that in *A. thaliana* leaves following DC3000 infection and prior to bacterial growth, photosynthesis is inhibited and chloroplastic ROS is suppressed (de Torres Zabala et al., 2015). Inhibition of photosynthesis would lead to the accumulation of NADP^+^ and one could envisage a mechanism would be needed to scavenge this NADP^+^. To address these concepts we speculated on a possible biosynthetic route for 3′-O-β-D-RFA (Fig. S20), either from AMP or NAD+ *via* activities of NUDIX hydrolase and phosphoribosyl/glycosyl transferases and subsequent phosphate removal, using an example of candidate gene expression profiles derived from published microarray data (Thilmony et al., 2006) to support our model. 3′-O-β-D-RFA's structure implicates NAD^+^ or NAADP may serve as a substrate not only for ADP-ribosylation reactions but also to generate precursors of the calcium mobilizing molecule cADPr (cyclic ADP-ribose). Alternatively 3′-O-β-D-RFA may function to dampen the pool of reducing equivalents which are essential to counteract oxidative damage and for other detoxifying reactions during host defense responses.

## 4 Experimental

### 4.1 *Arabidopsis thaliana* growth

Brassicaceae *A. thaliana* genotypes were sown in a 6:1 compost mix of Levingtons F2 compost with sand (LEV206): vermiculite (medium grade). Plants were grown under short days at 65% humidity in a controlled environment chamber (10 h light, 120 μE at 22 °C day, 20 °C night) for 4–5 weeks before use. Arabidopsis genotypes studied in this work were Col-0, *aao3* (de Torres Zabala et al., 2009) *coi1-*16 (Ellis and Turner, 2002) and *myc2* (Lorenzo et al., 2004).

Dexamethasone inducible HopAM1 lines in the Wassilewskija background (Goel et al., 2008) were treated with 5 μM of freshly prepared Dexamethasone (Sigma) and leaves harvested into liquid nitrogen at the appropriate timepoint.

### 4.2 *Pseudomonas syringae* infections

Bacterial cultures of the virulent Pseudomonadaceae *Pseudomonas syringae* pv. tomato strain DC3000 were maintained, prepared and inoculated in Kings B medium as described (de Torres et al., 2006). For metabolite extractions and growth curves (following 3′-O-β-D-RFA pre-treatment and co-infiltration), *A. thaliana* leaves were inoculated with a 1-ml needleless syringe on their abaxial surface with a bacterial suspension adjusted with 10 mM MgCl_2_ to a final density OD_600_ 0.15 (∼0.75 × 10^8^ colony forming units (cfu) ml^−1^) to measure 3′-O-β-D-RFA accumulation or OD_600_ 0.0002 to test impact of 3′-O-β-D-RFA pre-treatment (16 h pre-infiltration) and co-infiltration on bacterial growth. All bacterial growth measurements were determined from a minimum of 5 independent replicates, each comprising three challenged leaves/plant.

### 4.3 3′-O-β-D-ribofuranosyladenosine measurements

3'-O-β-D-RFA was measured by a modification of the method of (Forcat et al., 2008). Three-four leaves of fully developed leaves of 4–5 week old wild type *Arabidopsis thaliana* Col-0 or specific homozygous mutant lines (*aao3, coi1* or *myc2*) were challenged as described. Each replicate comprised of 6–8 leaves from 2 plants and three biological replicates per experiment were harvested at the specified specific time. Leaves were snap frozen in liquid nitrogen then freeze dried. 10 mg of freeze dried leaf powder was extracted with 400 μl ice cold extraction buffer (10% methanol, 0.1% acetic acid), containing unlabelled umbilliferone (Sigma, UK) as an internal standard (14.4 μg/sample). Samples were left on ice for 30 min with vortexing, then sonicated in an ice bath for 10 min. After centrifugation (10 min at 16,100×*g*, 4 °C), the pellet was re-extracted in 400 μl ice cold extraction buffer (without internal standard) and both supernatants pooled, then filtered through a 0.2 μm (PVDF) syringe filter (Chromacol, Welwyn Garden City, UK). 3′-O-β-D-RFA quantitative analysis was performed using an Agilent 6420B triple quadrupole (QQQ) mass spectrometer (Technologies, Palo Alto, USA) coupled to a 1200 series Rapid Resolution HPLC system. 10 μl of sample extract were loaded onto a Zorbax Eclipse Plus C18 (3.5 μm, 2.1  mm × 100 mm) reverse phase analytical column (Agilent Technologies, Palo Alto, USA). Samples were loaded in buffer A (1 mM ammonium fluoride, 5% acetonitrile) and separated at a flow rate of 0.3 ml/min^−1^ using Buffer B (95% acetonitrile) by one of two gradient protocols; t = 0 (5% B); t = 6min (100% B); t = 8min (100% B), t = 8.5 min (5% B) t = 12 min (5% B) or t = 0 (0% B); t = 5 min (20% B); t = 20 min (100% B); t = 25 min (100% B); t = 27 min (0% B).

Samples were detected in positive mode using the following transitions: 3′-O-β-D-RFA; 400 > 136 and 400 > 268; Umbelliferone; 163 > 107 and adenosine 268 > 136. The QQQ source conditions were as follows: gas temperature 350 °C, drying gas flow rate 9 l min-1, nebuliser pressure 35 psig, capillary voltage ±4 kV. For all samples the dwell time of 50, a fragmentor voltage of 90 and a collision energy of 20 V were used. Significance differences between treatments were determined by Students t-test (*p* < 0.5), error bars representing standard deviation of the mean. All experiments were repeated at least three times.

### 4.4 3′-O-β-D-ribofuranosyladenosine synthesis

Solvents and materials of reagent grade were used without additional purification. Column chromatography was performed on silica gel (Kieselgel 60, Merck, 0.040–0.063 mm) using EtOH—CH_2_Cl_2_ as eluent system. TLC was performed on TLC silica gel 60 F_254_ (Merck) with UV visualization. Melting points were determined on an Electrothermal apparatus and are uncorrected. ^1^H and ^13^C (with complete proton decoupling) NMR spectra were recorded on Bruker AMX 400 NMR and Bruker AVANCE II 300 instruments at 305 K. NMR spectra of compound **5** in D_2_O were recorded at elevated temperature (328 K) because of its low solubility in both water and organic solvents. Chemical shifts, δ, are given in ppm and measured relative to solvent signals (CDCl_3_, 1H: δ = 7.26, 13C: δ = 77.16; DMSO‑*d*_6_ 1H: δ = 2.50; CD_3_OD, 1H: δ = 3.31, 13C: δ = 49.00, D_2_O, 1H: δ = 4.79). Coupling constants, *J*, are given in hertz (Hz). Double resonance technique was applied to assign the resonances. UV-spectra were recorded on a Cary300 UV/VIS spectrophotometer (Varian). LC-MS analysis was performed on Surveyor MSQ instrument (Thermo Finnigan, USA), operating in APCI mode with detection of positive and negative ions, and equipped with an Onyx Monolithic C18 25 × 4.6 mm Part No CHO-7645 column; eluent: 0.1% HCOOH—H_2_O gradient in MeCN. Chromatographic peaks were detected simultaneously with ELSD, PAD and TIC detectors. 1-*O*-Acetyl-2,3,5-three-*O*-benzoyl-β-D-ribofuranose (**2**) was purchased from Pfanstiehl Laboratories Inc. (USA).

#### 4.4.1 Preparation of 2′,5′-di-O-tert-butyldimethylsilyladenosine (1)

2′,5′-Di-*O*-*tert*-butyldimethylsilyladenosine (**1**) was prepared according to the literature (Ogilvie et al., 1978). R_*f*_ 0.44 (toluene:ethyl acetate – 1:4). ^1^H-NMR (DMSO‑*d*_6_): 8.27 s (1H, H-8), 8.13 (1H, H-2), 7.26 s (2H, NH_2_-Ade), 5.94 s (1H, *J*_*1′,2’*_ = 5.2, H-1′), 5.07 d (1H, *J*_*3′,3′*__*-OH*_ = 5.6, 3′-OH), 4.63 dd (1H, *J*_*2′,3’*_ = 5.1, H-2′), 4.15 ddd (1H, *J*_*3′,4’*_ = 3.4, H-3′), 4.01 td (1H, *J*_*4′,5‘a*_ = *J*_*4′,5′*__*b*_ = 3.8, H-4′), 3.93 dd (1H, *J*_*5′a,5‘b*_ = - 11.4, H-5′a), 3.79 dd (1H, H-5′b), 0.90 s (9H, Me_3_C), 0.75 s (9H, Me_3_C), 0.08 s (3H, Me), 0.07 s (3H, Me), −0.06 s (3H, Me), −0.16 s (3H, Me). ^13^C-NMR (CDCl_3_): 155.33 (C-2), 152.60 (C-6), 150.06 (C-4), 139.29 (C-8), 119.93 (C-5), 88.24 (C-1′), 85.41 (C-4′), 71.34 (C-3′), 63.24 (C-5′), 26.17(Me_3_*C*), 25.73 (Me_3_*C*), 18.62, 18.08 (Me), −4.85, −5.15, −5.19, −5.31 (Me).

#### 4.4.2 Preparation of 9-[2,5-di-O-(tert-butyldimethylsilyl)-3-O-(2,3,5-three-O-benzoyl-β-D-ribofuranosyl)-β-D-ribofuranosyl]adenine (3)

To a cold solution (0°С) of 1-*O*-acetyl - 2,3,5-three-*O*-benzoyl-β-D-ribofuranose (**2**) (1.068 g, 2.12 mmol) in 1,2-dichloroethane (15 ml) under nitrogen tin tetrachloride (0.44 ml, 3.8 mmol) was added and the solution was kept at 0°С for 5 h. After addition of nucleoside **1** (700 mg, 1.4 mmol) the resulting solution was kept at 0°С for 16 h. Then 10% aqueous solution of sodium bicarbonate (50 ml) was added and the suspension was stirred at 0°С for 20 min. The suspension was diluted with methylene chloride (30 ml), filtered through *Hyflo Super Cel*, the organic layer was separated, washed with water (10 ml), dried over sodium sulfate and evaporated to dryness. The residue was purified by column chromatography on silica gel (50 g). The column was washed with methylene chloride (500 ml), and then eluted with 1% ethanol in methylene chloride to give **3** as a foam. Yield 842 mg (64%). R_*f*_ 0.31 (methylene chloride:ethanol – 98:2). ^1^H-NMR (CDCl_3_): 8.33 s (1H, H-8), 8.30 s (1H, H-2), 7.99 dd (2H, *J*_o-H, m-H_ = 8.3, *J*_o-H, p-H_ = 1.1, Bz), 7.95 dd (2H, *J*_o-H, m-H_ = 8.4, *J*_o-H, p-H_ = 1.2, Bz), 7.91 dd (2H, *J*_o-H, m-H_ = 8.2, *J*_oH, p-H_ = 1.2, Bz), 7.58 tt (1H, *J*_p-H, m-H_ = 6.1, *J*_p-H, o-H_ = 1.2, Bz), 7.55–7.48 m (2H, Bz), 7.42 dd (2H, *J*_m-H, o-H_ = 8.2, *J*_m-H, p-H_ = 6.1, Bz), 7.38–7.30 m (4H, Bz), 6.34 br.s (2H, NH_2_-Ade), 6.06 d (1H, *J*_*1′,2’*_ = 3.4, H-1′ Ado), 5.75 dd (1H, *J*_*3′,2’*_ = 5.1, *J*_*3′,4’*_ = 6.4, H-3′ Rib), 5.64 dd (1H, *J*_*2′,1’*_ = 1.4, H-2′ Rib), 5.45 d (1H, H-1′ Rib), 4.77 dd (1H, *J*_*2′,3’*_ = 4.0, H-2′ Ado), 4.72–4.61 m (2H, H-3′ Ado, H-4′ Rib), 4.60–4.52 m (2H, H-5′a, H-5′b Rib), 4.38 ddd (1H, *J*_*4′,3’*_ = 5.4, *J*_*4′,5′*a_ = 3.1, *J*_*4′,5‘*b_ = 2.6, H-4′ Ado), 4.12 dd (1H, *J*_*5′*__a*,5‘*b_ = −11.8, H-5′a Ado), 3.90 dd (1H, H-5′b Ado), 0.91 s (9H, Me_3_C), 0.89 s (9H, Me_3_C), 0.13 s (3H, Me), 0.11 s (3H, Me), 0.09 s (3H, Me), 0.00 s (3H, Me). ^13^C-NMR (CDCl_3_): 166.18, 165.40, 165.37 (C=O), 155.54 (C-2), 153.15 (C-6), 149.95 (C-4), 139.31 (C-8), 133.55, 133.22, 129.91, 129.88, 128.62, 128.50, 128.46 (Bz), 120.29 (C-5), 104.66 (C-1′ Rib), 89.16 (C-1′ Ado), 82.08 (C-4′ Ado), 79.17 (C-3′ Ado), 75.65 (C-4′ Rib), 75.57 (C-2′ Rib), 75.33 (C-2′ Ado), 72.50 (C-3′ Rib), 65.63 (C-5′ Rib), 62.33 (C-5′ Ado), 26.09, 25.78 (Si*Me*_2_*t*-Bu), 18.50, 18.17 (*C*Me_3_), −4.66, −4.79, −5.23, −5.39 (C*Me*_3_).

#### 4.4.3 Preparation of 9-[3-O-(2,3,5-three-O-benzoyl-β-D-ribofuranosyl)-β-D-ribofuranosyl]adenine (**4**)

Nucleoside **3** (750 mg, 0.8 mmol) was dissolved in 0.5 M tetrabutylammonium fluoride trihydrate in tetrahydrofuran (2.2 ml). The solution was kept for 1 h at 20°С, evaporated to dryness, coevaporated with chloroform (2 × 15 ml) and applied onto silica gel column (20 g). The column was washed with methylene chloride (300 ml) and with 2% ethanol in methylene chloride (200 ml) and then eluted with 3.5% ethanol in methylene chloride to give **4** as a foam. Yield 313 mg (55%). R_*f*_ 0.41 (methylene chloride:ethanol – 95:5). ^1^H-NMR (CDCl_3_): 8.15 s (1H, H-2), 8.07 d (2H, *J*_o-H, m-H_ = 7.4, Bz), 7.99 d (2H, *J*_o-H, m-H_ = 7.4, Bz), 7.91 d (2H, *J*_o-H, m-H_ = 7.6, Bz), 7.70 s (H-8), 7.57 t (2H, *J*_m-H, o-H_ = *J*_m-H, p-H_ = 7.4, Bz), 7.54–7.49 m (1H, Bz), 7.41 t (4H, *J*_m-H, o-H_ = *J*_m-H,-p-H_ = 7.6, Bz), 7.34 t (2H, *J*_p-H, m-H_ = 7.6, Bz), 6.52 br.s (2H, NH_2_ Ade), 5.92 dd (1H, *J*_*3′,2’*_ = 4.7, *J*_*3′,4’*_ = 6.3, H-3′ Rib), 5.80 d (1H, H-2′ Rib), 5.74 d (1H, *J*_*1′,2’*_ = 7.2, H-1′ Ado), 5.51 s (1H, H-1′ Rib), 5.05 dd (1H, *J*_*2′,3’*_ = 4.8, H-2′ Ado), 4.85–4.71 m (3H, H-4′ Rib, H-5′a, H-5′b Rib), 4.66 d (1H, H-3′ Ado), 4.37 s (1H, H-4′ Ado), 3.97 d (1H, *J*_*5′*__a*,5′*b_ = −12.8, H-5′a Ado), 3.76 d (1H, H-5′b Ado). ^13^C-NMR (CDCl_3_): 166.50, 165.54, 165.43 (C=O), 155.58 (C-2), 151.75 (C-6), 148.45 (C-4), 141.10 (C-8), 133.82, 133.72, 133.60, 129.94, 129.90, 128.69, 128.58 (Bz), 120.85 (C-5), 106.36 (C-1′ Rib), 91.25 (C-1′ Ado), 85.88 (C-4′ Ado), 80.72 (C-3′ Ado), 80.05 (C-4′ Rib), 75.90 (C-2′ Rib), 73.54 (C-2′ Ado), 72.08 (C-3′ Rib), 64.83 (C-5′ Rib), 63.02 (C-5′ Ado). LC-MS (APCI): retention time - 2.35 min; m/*z* (rel. int): [M+H]^+^ 712.59 (100).

#### 4.4.4 Preparation of 9-[3-О-β-D-ribofuranosyl-β-D- ribofuranosyl]adenine (**5**)

A solution of nucleoside **4** (300 mg, 0.421 mmol) in 5 M ammonia in methanol (16 ml) was kept for 5 days at 20°С and then concentrated in vacuo to dryness. The residue was partitioned between chloroform (10 ml) and water. The aqueous layer was washed with chloroform (4 × 15 ml). The aqueous layer was evaporated to the volume ∼2 ml and was left to stay overnight at 0°С. The precipitate was filtered, washed with acetone (2 × 5 ml) and dried in vacuum desiccator over phosphorus pentoxide. Yield 110 mg (65%) as a white powder. M.p. 232–234°С (dec.). R_*f*_ 0.075 (methylene chloride:ethanol – 80:20). UV (H_2_O): *λ*_max_, nm (*ε*, M^−1^cm^−1^): 259 (14900) (pH 7–13), 257 (14500) (pH 2). ^1^H-NMR (D_2_O): 8.39 s (1H, H-2), 8.30 s (1H, H-2), 6.14 d (1H, *J*_*1′,2’*_ = 5.0, H-1′ Ado), 5.28 s (1H, H-1′ Rib), 4.91 dd (1H, *J*_*2′,3’*_ = 4.9, H-2′ Ado), 4.58 dd (1H, *J*_*3′,4’*_ = 4.8, H-3′ Ado), 4.49–4.42 m (2H, H-3′ Rib, H-4′ Ado), 4.29 d (1H, *J*_*2′,3’*_ = 4.7, H-2′ Rib), 4.20–4.14 m (1H, H-4′ Rib), 4.03 dd (1H, *J*_*5′*__a*,4’*_ = 2.8, *J*_*5′*__a*,5′*b_ = −12.9, H-5′a Rib), 3.95 dd (1H, *J*_*5′*__a*,4’*_ = 2.6, *J*_*5′*__a*,5′*b_ = −12.5, H-5′a Ado), 3.91 dd (1H, *J*_*5′*__b*,4’*_ = 3.6, H-5′b Rib), 3.82 dd (1H, *J*_*5′*__b*,4’*_ = 5.1, H-5′b Ado). ^13^C-NMR (D_2_O, 323 K): 155.92 (C-8), 143.57 (C-2), 123.00 (C-5), 110.01 (C-1′ Rib), 91.65 (C-1′ Ado), 86.40, 86.27 (C-4′ Ado, C-4′ Rib), 79.72 (C-3′ Ado), 77.90 (C-2′ Rib), 76.33 (C-2′ Ado), 73.11 (C-3′ Rib), 64.57, 64.25 (C-5′ Ado, C-5′ Rib). LC-MS (APCI): retention time – 0.44 min; m/*z* (rel. int): [M+H]^+^ 400.28 (100).

## 5 Conclusion

We characterize the dynamics of accumulation of the natural disaccharide nucleoside, 3′-O-β-D-ribofuranosyladenosine, in *Arabidopsis thaliana* leaves infected with virulent DC3000 and show it is a plant specific molecule and its increase is directly correlated to bacterial multiplication. We provide a detailed method for the synthesis of 3′-O-β-D-ribofuranosyladenosine and its detailed characterization. While this allowed the quantification of the accumulation of 3′-O-β-D-ribofuranosyladenosine in infected leaves, including its exponential increase during the latter stages of infection. We speculate a role for 3′-O-β-D-ribofuranosyladenosine in altering the hosts pool of pyridine nucleotides to suppress plant defense responses. However, we were unable to demonstrate any biological activity - either in modulating bacterial growth or modifying bacterial load in infected leaves. Future work will explore the possibility whether 3′-O-β-D-ribofuranosyladenosine derivatives are biologically active compounds, or 3′-O-β-D-ribofuranosyladenosine's key role is to deplete the plant pool of pyridine nucleotides.

## Funding sources

The chemistry (synthesis and characterization of RFA) was supported by the 10.13039/501100006769Russian Science Foundation (project №17-74-10057) and the biology and plant pathology work was supported by British Biotechnology and Science Research Council grants (BB/F005903/1 & BB/P002560/1) to MG.
