# Supplementary material for: A role for 3′-O-β-D-ribofuranosyladenosine in altering plant immunity
Source: Phytochemistry. 2019 Jan;157:128–34. doi: 10.1016/j.phytochem.2018.10.016 (PMC6290457; doi:10.1016/j.phytochem.2018.10.016)
Supplement: Multimedia component 1 [file mmc1.docx]

**Supplementary Material: A role for 3'-O-β-D-ribofuranosyladenosine in altering plant immunity**

Mikhail S. Drenichev^1a^, Mark Bennett^2a^, Roman A. Novikov^1^, John Mansfield^2^, Nick Smirnoff^3^, Murray Grant^4^* and Sergey N. Mikhailov^1^*

^a^ both authors contribute equally.

*authors for correspondence

^1^Engelhardt Institute of Molecular Biology, Russian Academy of Sciences, Vavilov str. 32, Moscow 119991, Russian Federation

^2^Imperial College London, Exhibition Road, *London* SW7 2AZ, United Kingdom

^3^School of Biosciences, University of Exeter, Stocker Road, Exeter, EX4 4QD, United Kingdom

^4^School of Life Sciences, Gibbet Hill, University of Warwick, Coventry, CV4 7AL, United Kingdom

**Figure S1** Foliar accumulation of 3'-O-β-D-ribofuranosyladenosine following infection with virulent *P. syringae* DC3000 (OD_600_ 0.15 OD). Samples were snap frozen at the times indicated and 3'-O-β-D-ribofuranosyladenosine measured as described in methods. Error bars represent the standard deviation of the mean.

**Figure S2.** Foliar accumulation of 3'-O-β-D-ribofuranosyladenosine inducted by the activity of a single bacterial effector, HopAM1. Leaves of a transgenic *A. thaliana*  Ws-0 lines conditionally expressing *HopAM1* from a dexamethasone inducible promoter showed accumulation of 3'-O-β-D-ribofuranosyladenosine within 12h of dexamethasone (5 μM) application. By contrast 3'-O-β-D-RFA did not accumulate above basal levels in wild type *A. thaliana*  Ws-0 . Dexamethasone treated leaves were snap frozen at the times indicated and 3'-O-β-D-ribofuranosyladenosine measured as described in experimental methods.

**Figures S3-S19**

The solvents and materials of reagent grade were used without additional purification. Column chromatography was performed on silica gel (Kieselgel 60, Merck, 0.040 – 0.063 mm) using EtOH-CH_2_Cl_2_ as eluent system. TLC was performed on TLC silica gel 60 F_254_ (Merck) with UV visualization. Melting points were determined on a Electrothermal apparatus and are uncorrected. ^1^H and ^13^C (with complete proton decoupling) NMR spectra were recorded on Bruker AMX 400 NMR (^1^H: 400.1 MHz, ^13^C: 100.6 MHz) and Bruker AVANCE II 300 (^1^H: 300.1 MHz, ^13^C: 75.5 MHz) instruments at 305 K. NMR spectra of compound **5** in D_2_O were recorded at elevated temperature (328K) because of its low solubility both in both water and organic solvents. Chemical shifts, δ, are given in ppm and measured relative to solvent signals (CDCl_3_, 1H: δ = 7.26, 13C: δ = 77.16; DMSO-*d*_6_ 1H: δ = 2.50; CD_3_OD, 1H: δ = 3.31, 13C: δ = 49.00, D_2_O, 1H: δ = 4.79). Coupling constants, *J*, are given in hertz (Hz). Double resonance technique was applied to assign the resonances. UV-spectra were recordered on a Cary300 UV/VIS spectrophotometer (Varian). LC-MS analysis was performed on Surveyor MSQ instrument (Thermo Finnigan, USA), operating in APCI mode with detection of positive and negative ions, and equipped with an Onyx Monolithic C18 25×4.6 mm Part No CHO-7645 column; eluent: 0.1% HCOOH-H_2_O gradient in MeCN. Chromatographic peaks were detected simultaneously with ELSD, PAD and TIC detectors.

**Figure S3.** ^1^H-NMR spectrum (400.1 MHz) of 2',5'-Di-*O*-*tert*-butyldimethylsilyladenosine (**1**) in DMSO-*d*_6_ at 305K


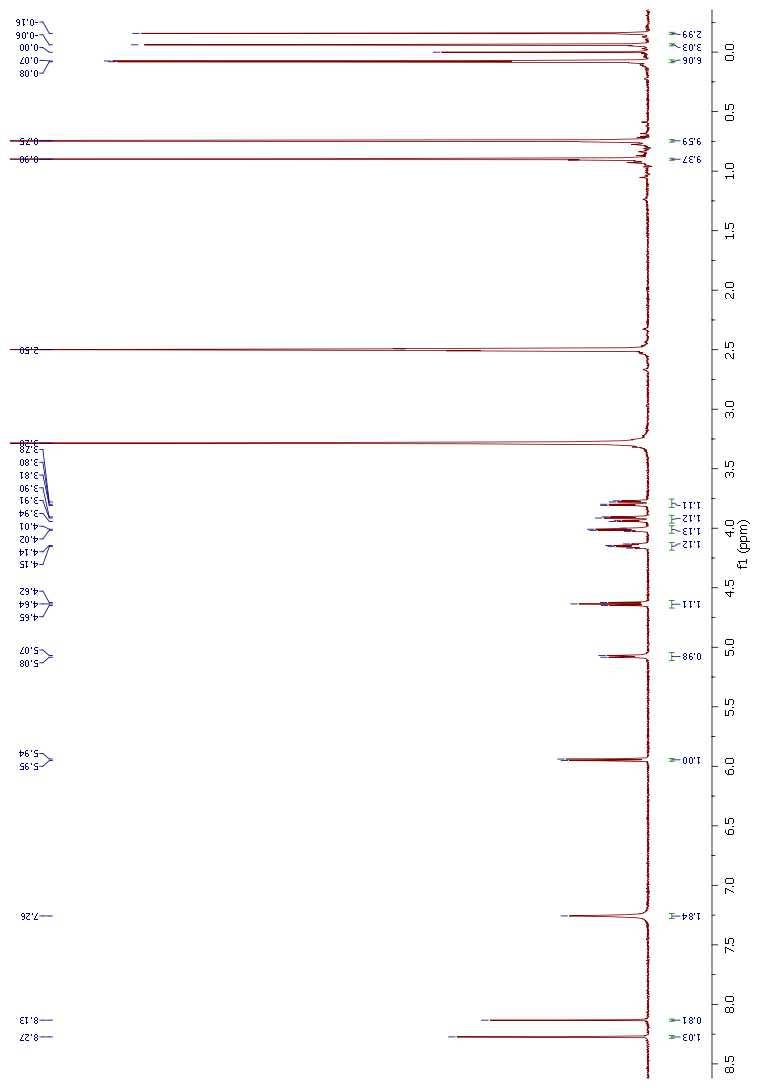


**Figure S4.** ^13^C-NMR spectrum (100.6 MHz) of 2',5'-Di-*O*-*tert*-butyldimethylsilyladenosine (**1**) in CDCl_3_ at 305K


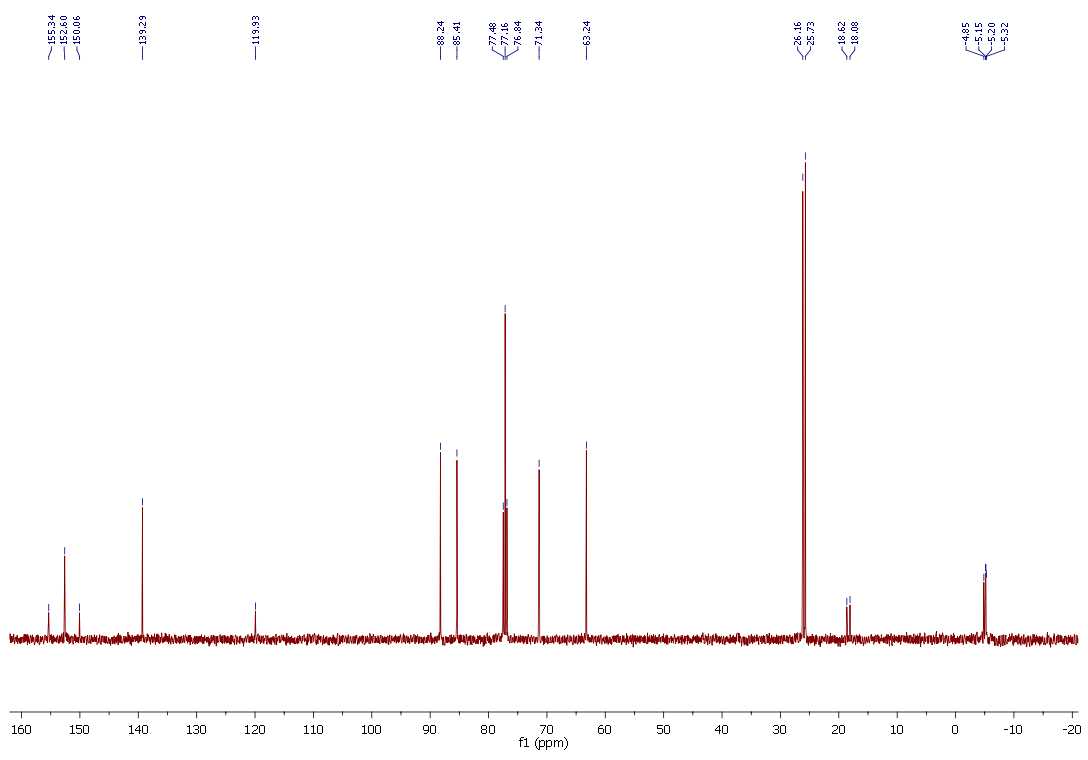


**Figure S5**. ^1^H-NMR spectrum (400.1 MHz) of 9-[2,5-Di-*O*-(*tert*-butyldimethylsilyl)-3-*O*-(2,3,5-tri-*O-*benzoyl-β-D-ribofuranosyl)-β-D-ribofuranosyl]adenine (**3**) in CDCl_3_ at 305K


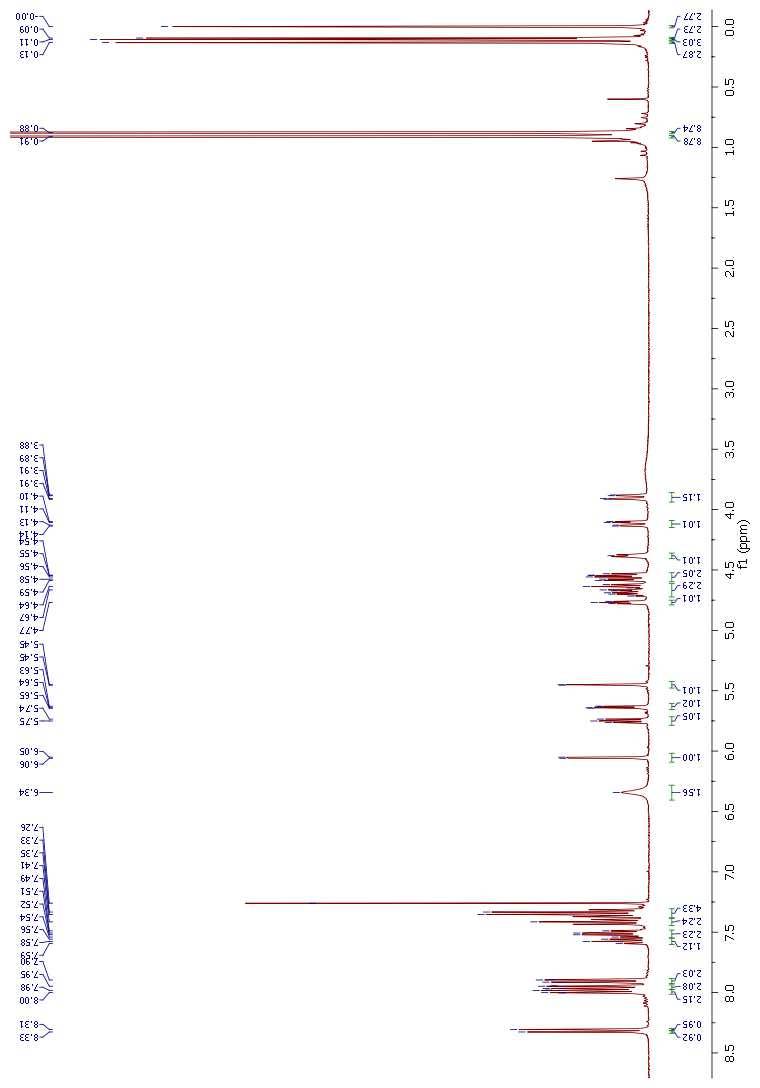


**Figure S6.** ^13^C-NMR spectrum (100.6 MHz) of 9-[2,5-Di-*O*-(*tert*-butyldimethylsilyl)-3-*O*-(2,3,5-tri-*O-*benzoyl-β-D-ribofuranosyl)-β-D-ribofuranosyl]adenine (**3**) in CDCl_3_ at 305K

+
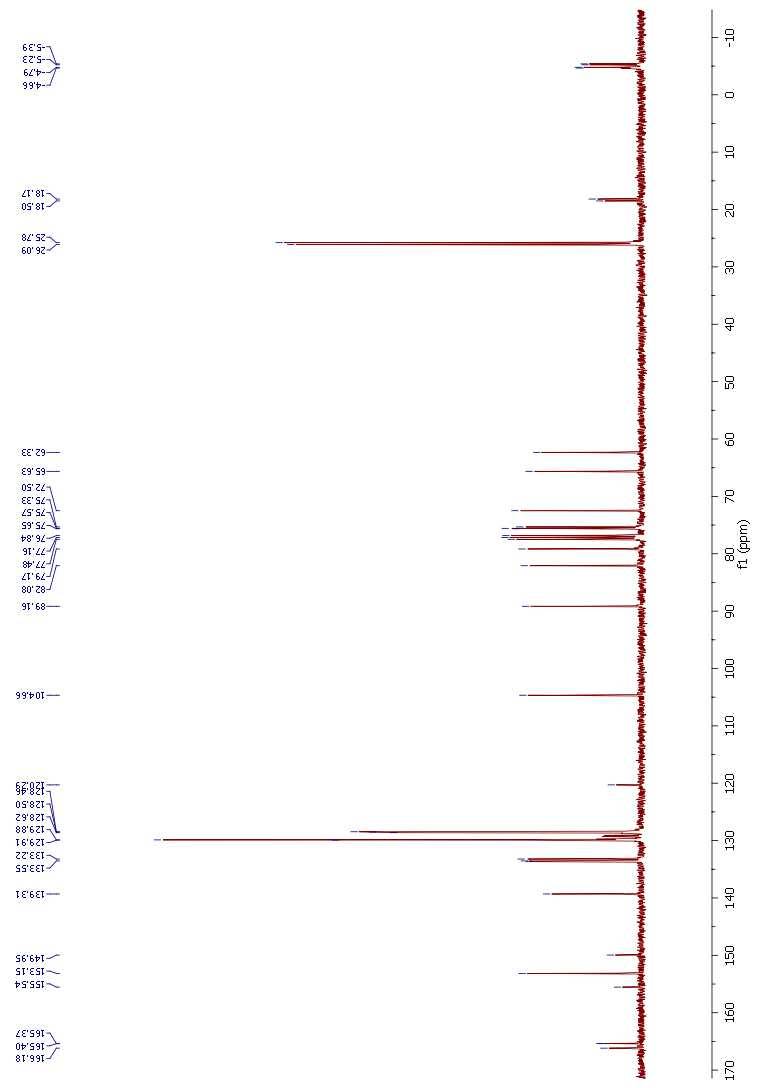


**Figure S7.** ^1^H-NMR spectrum (300.1 MHz) of 9-[3-*O*-(2,3,5-tri-*O-*benzoyl-β-D-ribofuranosyl)-β-D-ribofuranosyl]adenine (**4**) in CDCl_3_ at 305K

**
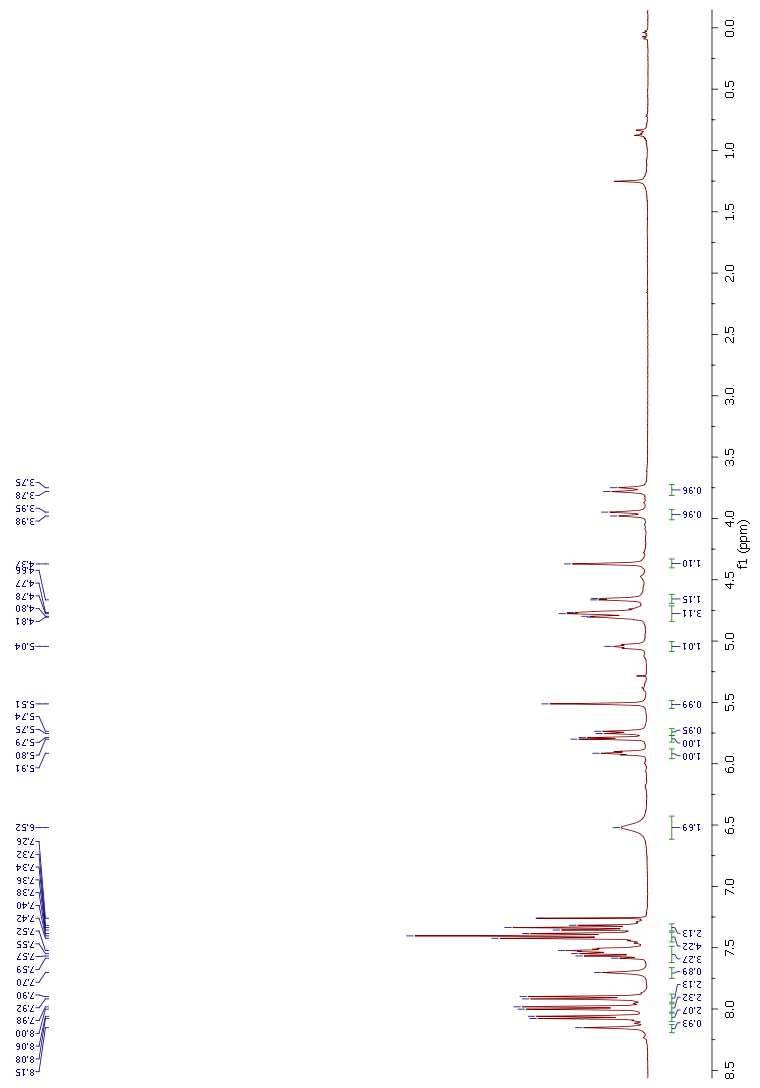
**

**Figure S8.** ^13^C-NMR spectrum (75.5 MHz) of 9-[3-*O*-(2,3,5-tri-*O-*benzoyl-β-D-ribofuranosyl)-β-D-ribofuranosyl]adenine (**4**) in CDCl_3_ at 305K


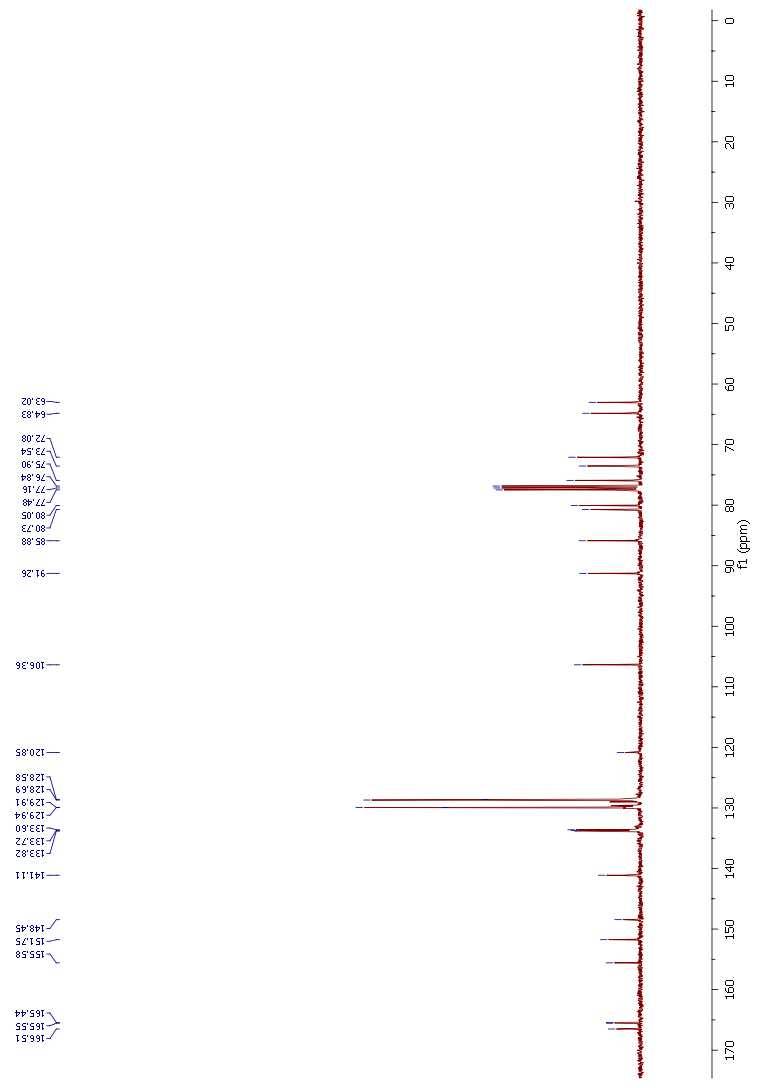


**Figure S9.** HSQC spectrum of 9-[3-*O*-(2,3,5-tri-*O-*benzoyl-β-D-ribofuranosyl)-β-D-ribofuranosyl]adenine (**4**) in CDCl_3_ at 305K (^1^H: 300.1 MHz, ^13^C: 75.5 MHz)

^
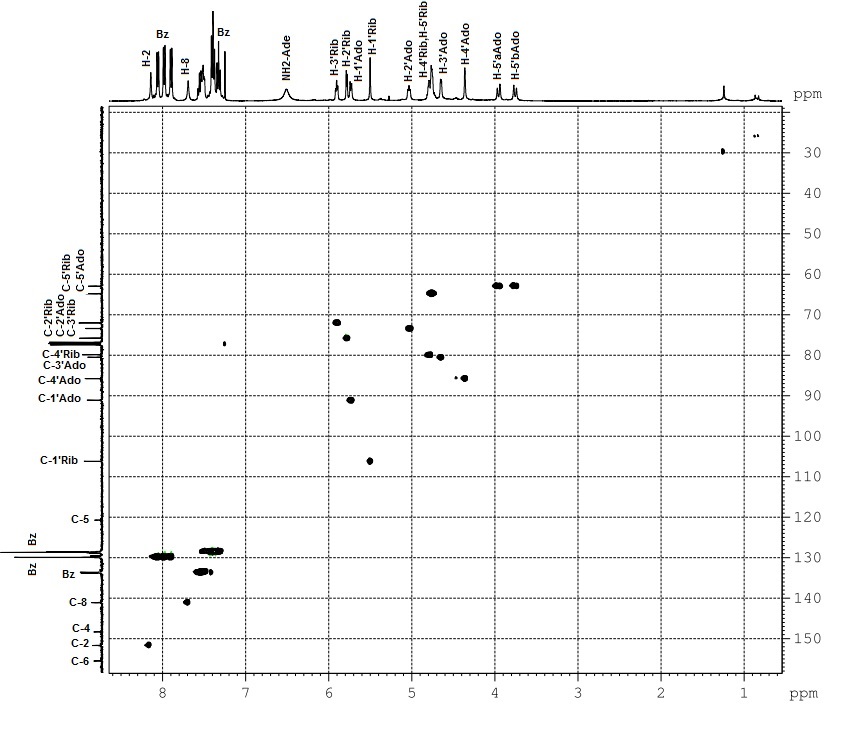
^

**Figure S10.** HMBC spectrum of 9-[3-*O*-(2,3,5-tri-*O-*benzoyl-β-D-ribofuranosyl)-β-D-ribofuranosyl]adenine (**4**) in CDCl_3_ at 305K (^1^H: 300.1 MHz, ^13^C: 75.5 MHz)


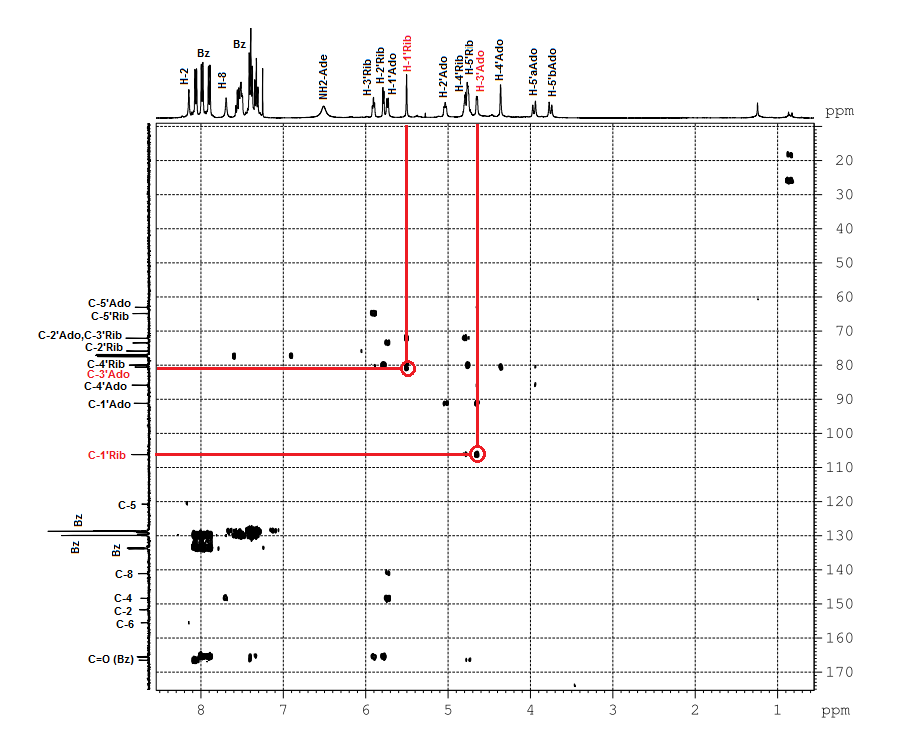


**Figure S11.** MS-spectrum (APCI) of 9-[3-*O*-(2,3,5-tri-*O-*benzoyl-β-D-ribofuranosyl)-β-D-ribofuranosyl]adenine (**4**)

^
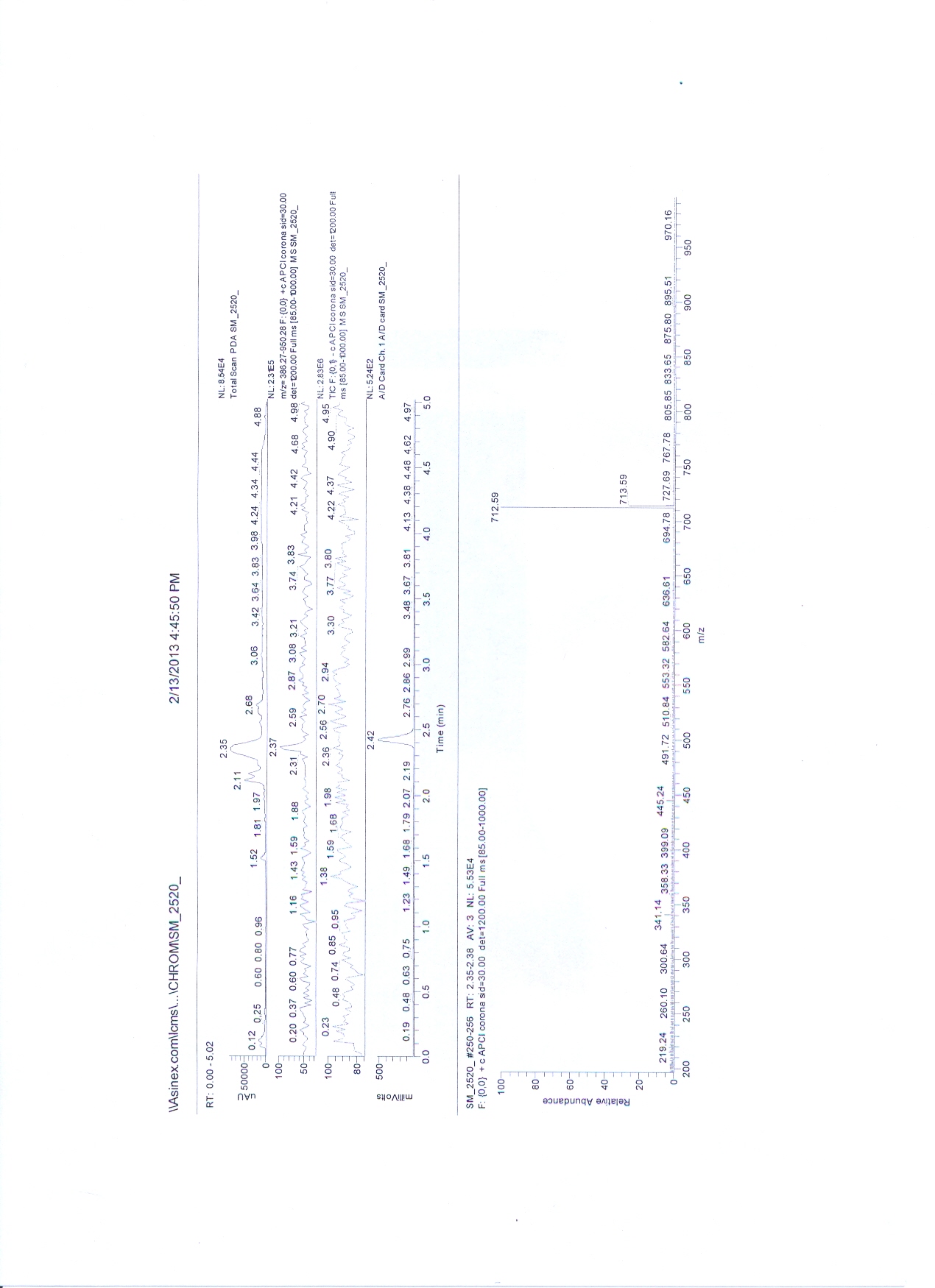
^

**Figure S12.** ^1^H-NMR spectrum (400 MHz) of 9-[3-*О*-β-D-ribofuranosyl-β-D- ribofuranosyl]adenine (**5**) in CD_3_OD at 305K


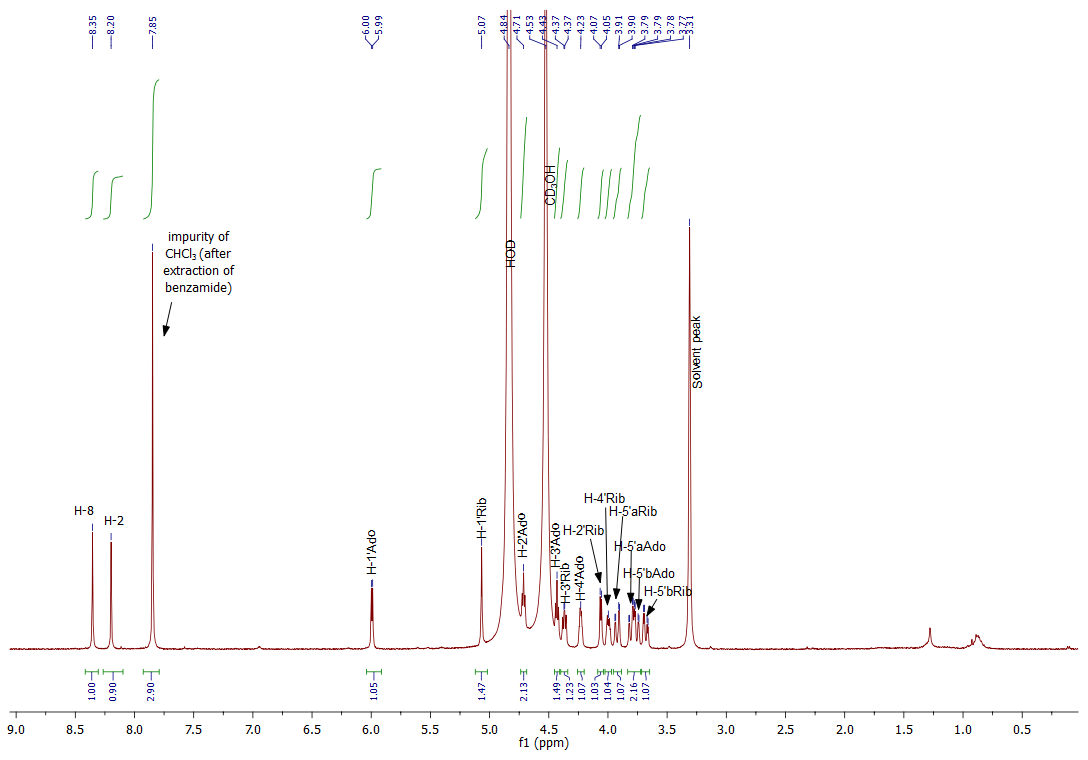


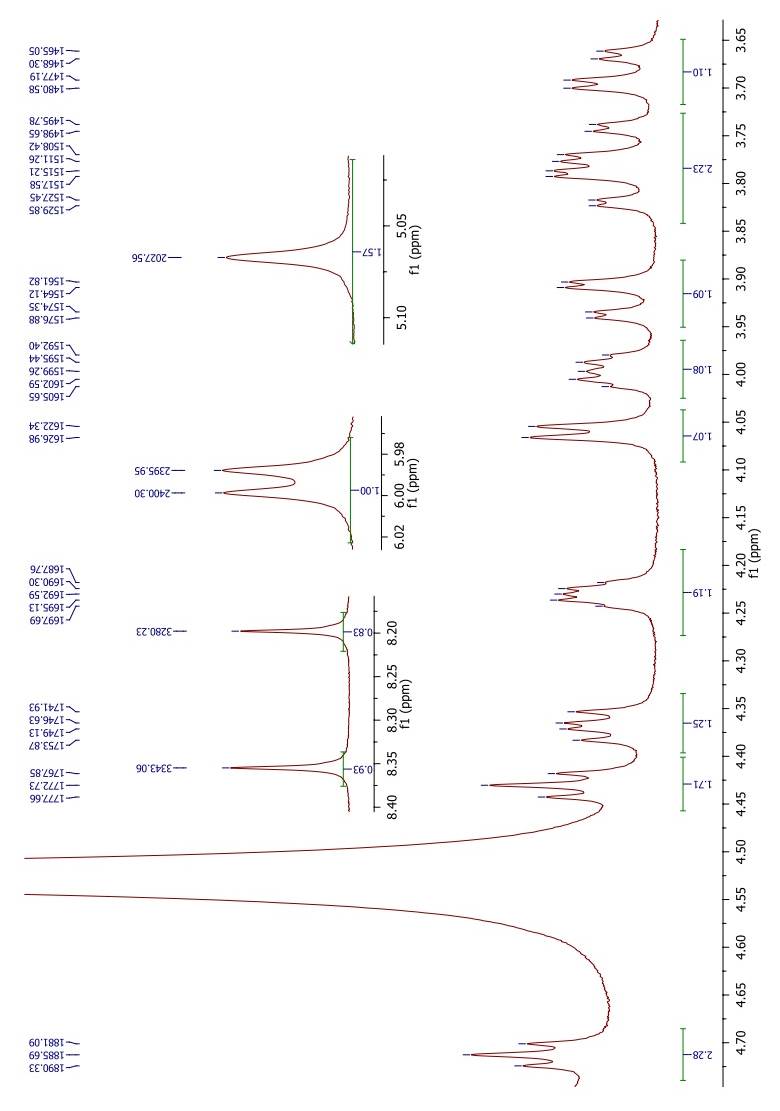


**Figure S13.** ^1^H-NMR spectrum (400.1 MHz) of 9-[3-*О*-β-D-ribofuranosyl-β-D- ribofuranosyl]adenine (**5**) in D_2_O at 305K


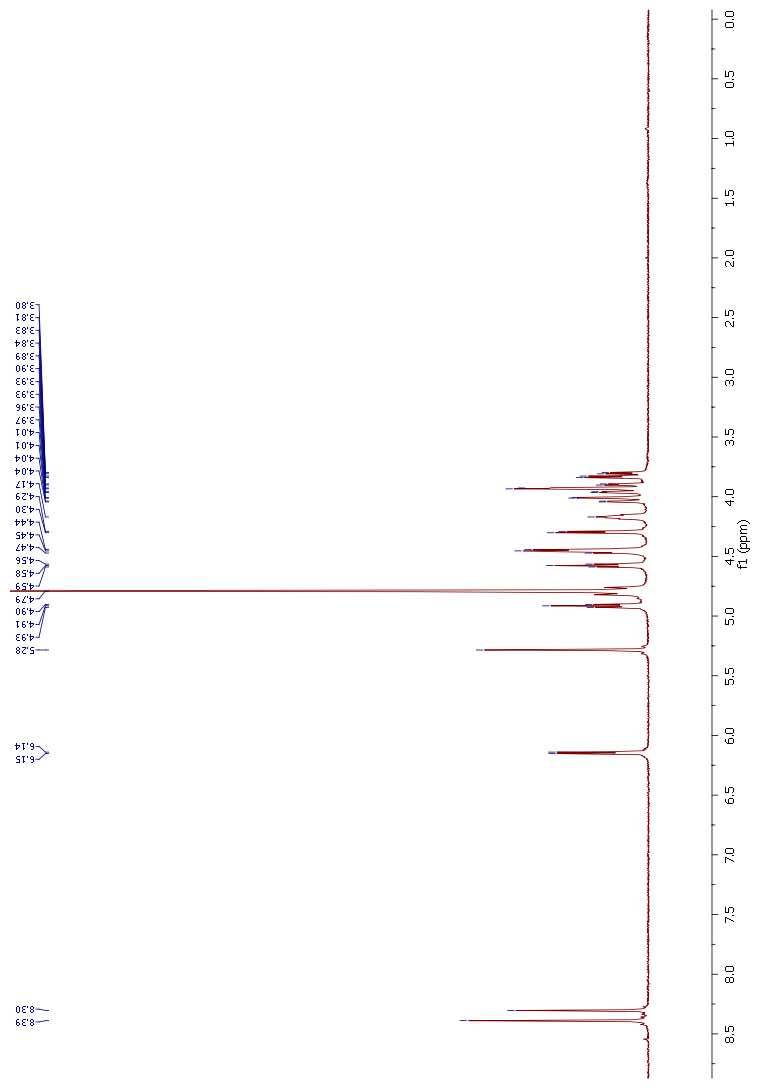


**Figure S14.** ^1^H-NMR spectrum (400.1 MHz) of 9-[3-*О*-β-D-ribofuranosyl-β-D- ribofuranosyl]adenine (**5**) in D_2_O at 328K

**
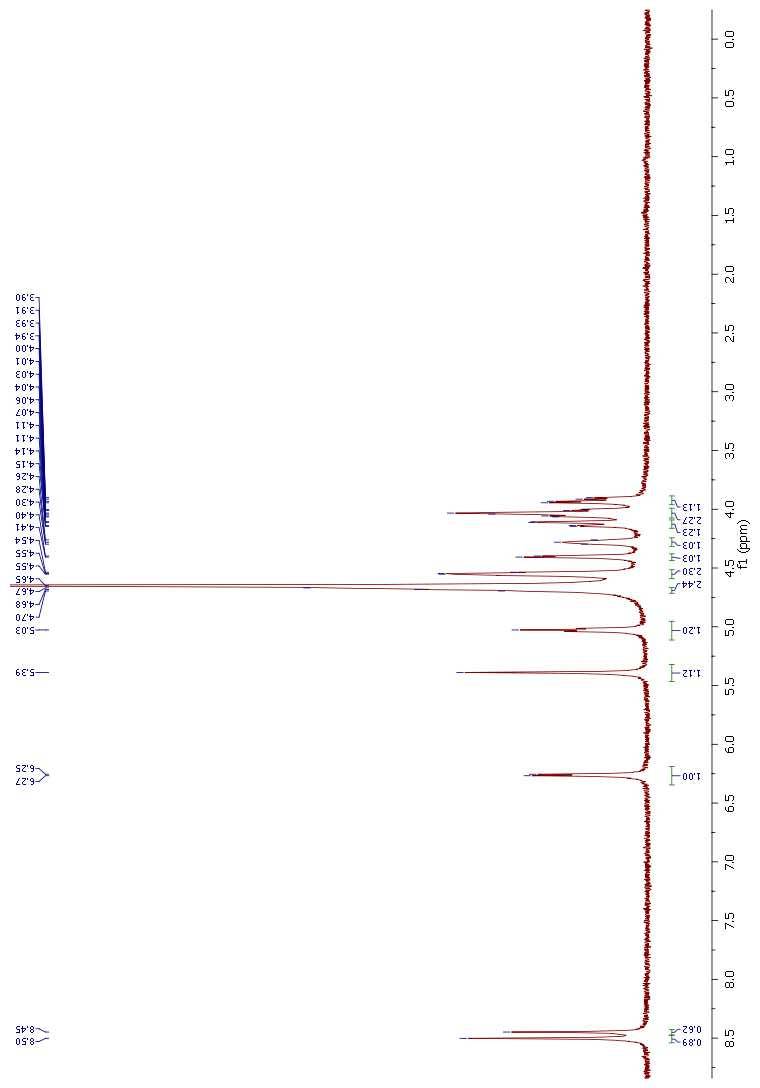
**

**Figure S15.** ^13^C-NMR spectrum (100.6 MHz) of 9-[3-*О*-β-D-ribofuranosyl-β-D- ribofuranosyl]adenine (**5**) in D_2_O at 328K (concentration of disaccharide nucleoside 3mg/0.6 ml)


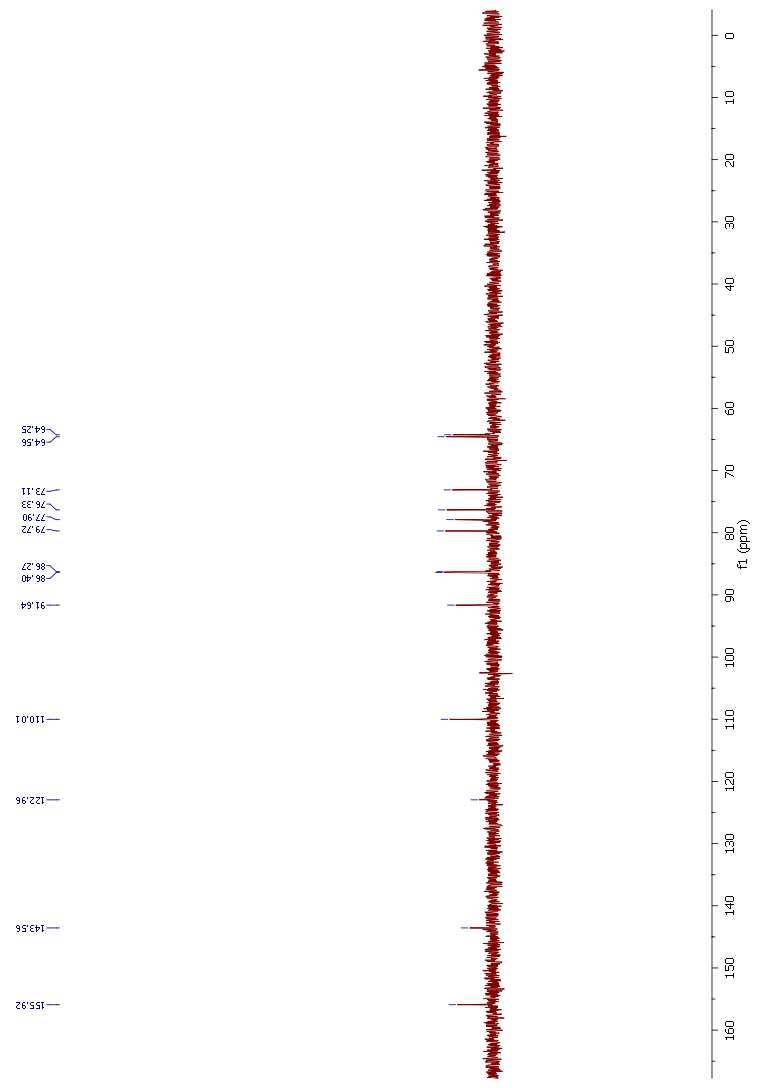


**Figure S16.** HSQC of 9-[3-*О*-β-D-ribofuranosyl-β-D- ribofuranosyl]adenine (**5**) in D_2_O at 328K (^1^H: 400.1 MHz, ^13^C: 100.6 MHz)


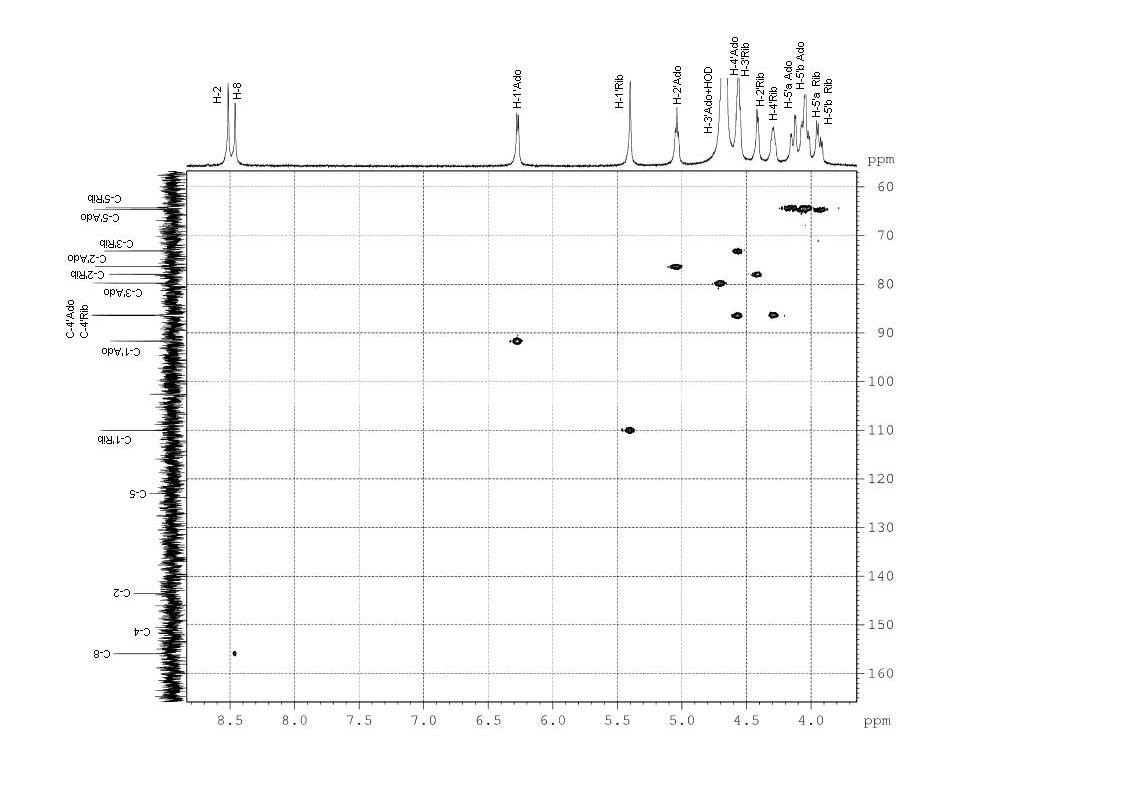


**Figure S17**. HMBC of 9-[3-*О*-β-D-ribofuranosyl-β-D- ribofuranosyl]adenine (**5**) in D_2_O at 328K (^1^H: 400.1 MHz, ^13^C: 100.6 MHz)


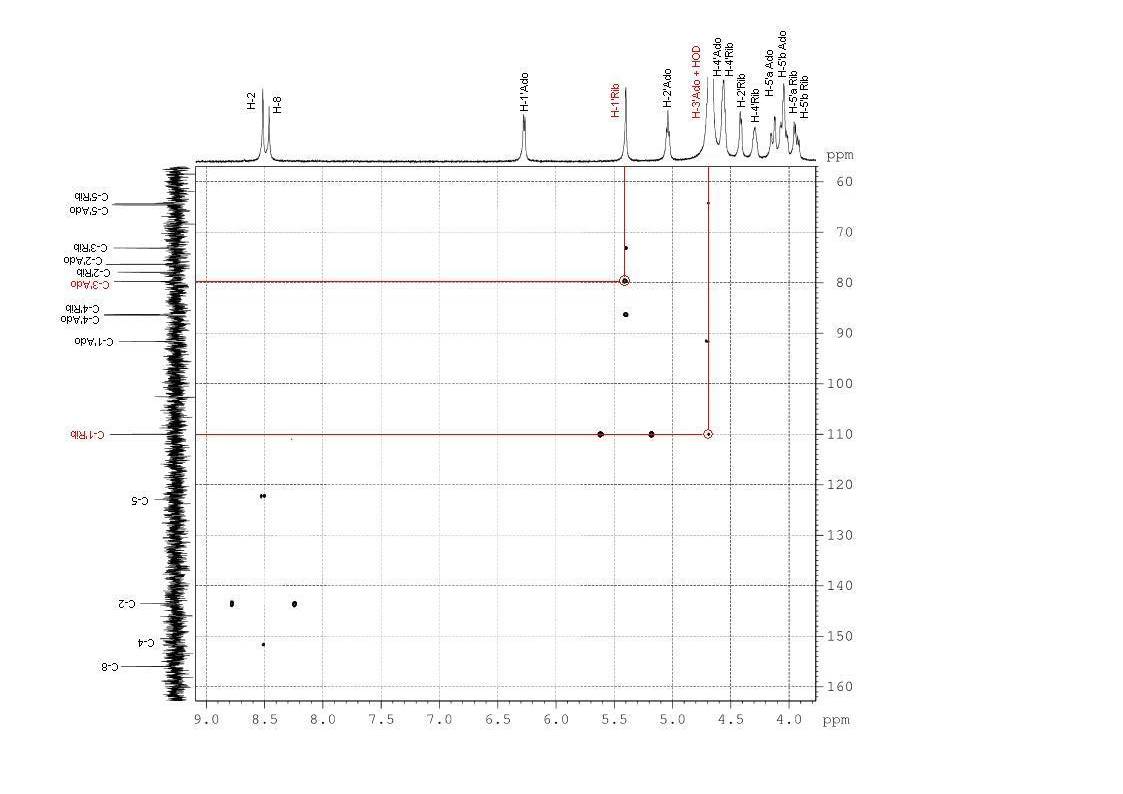
**Figure S18.** MS-spectrum (APCI) of 9-[3-*О*-β-D-ribofuranosyl-β-D- ribofuranosyl]adenine (**5**)


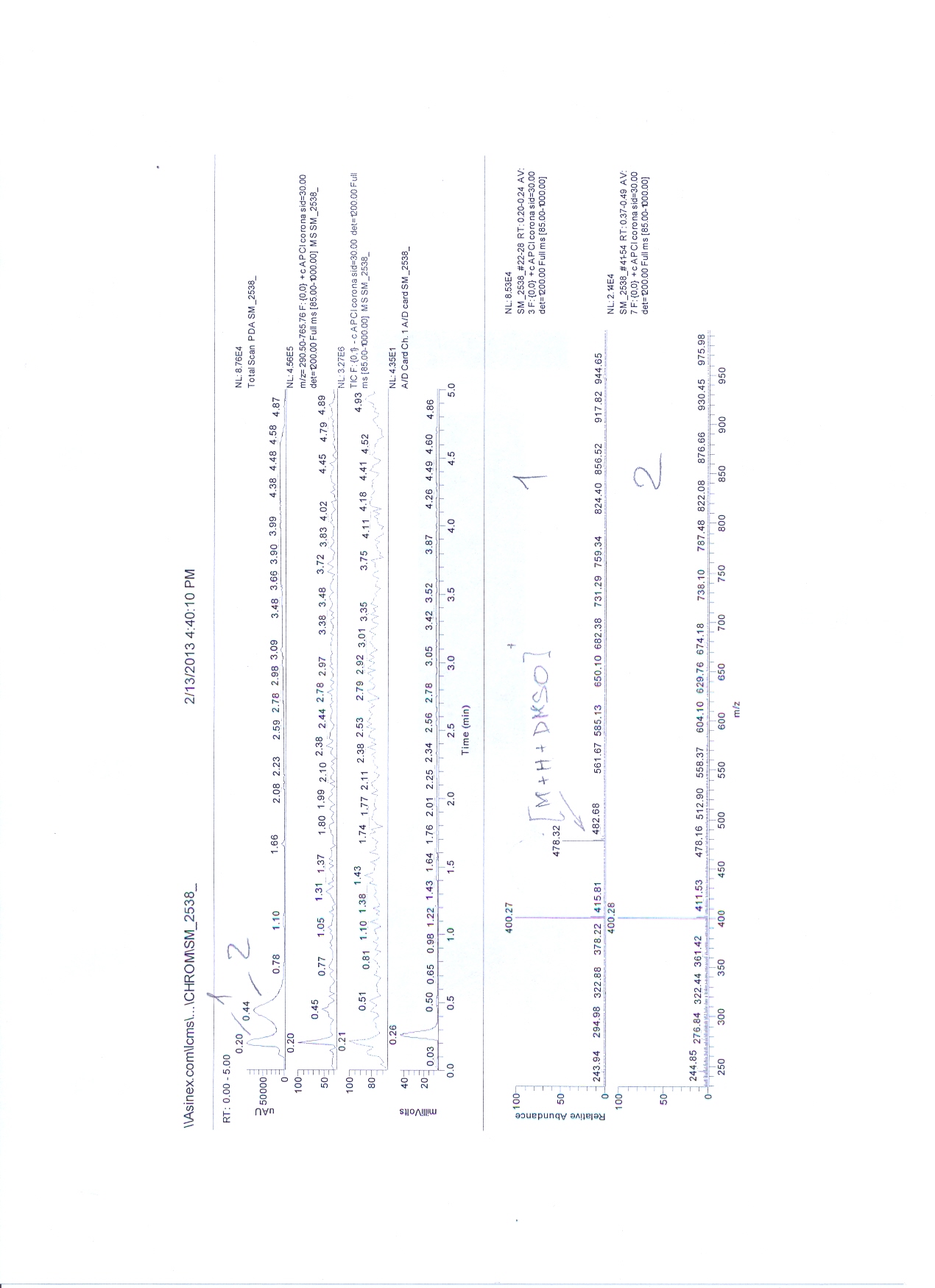


**Figure S19.** Molar absorbance of 9-[3-*О*-β-D-ribofuranosyl-β-D- ribofuranosyl]adenine (**5**) in aquous solution at various pH

*UV-spectrum of 3'-O-β-D-RFA in H_2_O is characterized by absorption maximum at 259 nm (ε ~14900) at рН 7-13 with slightly hypsochromic shift under acidic conditions (Figure S18), which is typical for adenine derivatives with substituents at position 9 of heterocycle* ( Albert, A. (1973) The Ultraviolet spectra of pyrimidines and purines. In: Physical and physicochemical aids in characterization and in determination of structure. (Zorbach, W. W. and R.S., T., eds.). New York: Wiley-Interscience, pp. 47-123*,* Dowson, R. M. C., Elliott, D. C., Elliott, W. H. and Jones, K. M. (1986) Data for biochemical research. Oxford: Oxford Science Publications*)*


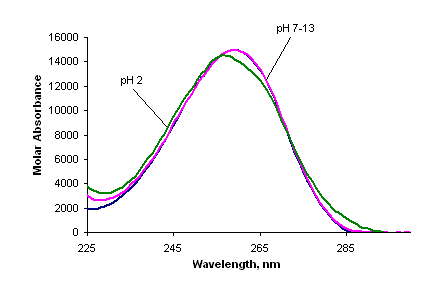


**Figure S20** Predicted route for synthesis of 3'-O-β-D-ribofuranosyladenosine, either from AMP or NAD+ via activities of nudix hydrolase and phosphoribosyl/glycosyl transferases followed by phosphate removal. Examples of expression profiles of candidates genes encoding enzymes involved in this hypothetical synthesis route that are specifically induced by DC3000 but not non-pathogenic bacteria within 7h of inoculation (Affymetrix probe set expression data derived from (Thilmony *et al.*, 2006).

Thilmony, R., Underwood, W. and He, S. Y. (2006) Genome-wide transcriptional analysis of the Arabidopsis thaliana interaction with the plant pathogen Pseudomonas syringae pv. tomato DC3000 and the human pathogen Escherichia coli O157:H7. *Plant J,* **46,** 34-53.
